# A direct-to-biology high-throughput chemistry approach to reactive fragment screening†

**DOI:** 10.1039/d1sc03551g

**Published:** 2021-08-11

**Authors:** Ross P. Thomas, Rachel E. Heap, Francesca Zappacosta, Emma K. Grant, Peter Pogány, Stephen Besley, David J. Fallon, Michael M. Hann, David House, Nicholas C. O. Tomkinson, Jacob T. Bush

**Affiliations:** GlaxoSmithKline Gunnels Wood Road Stevenage Hertfordshire SG1 2NY UK jacob.x.bush@gsk.com; Department of Pure and Applied Chemistry, University of Strathclyde 295 Cathedral Street Glasgow G1 1XL UK; GlaxoSmithKline South Collegeville Road Collegeville PA 19426 USA

## Abstract

Methods for rapid identification of chemical tools are essential for the validation of emerging targets and to provide medicinal chemistry starting points for the development of new medicines. Here, we report a screening platform that combines ‘direct-to-biology’ high-throughput chemistry (D2B-HTC) with photoreactive fragments. The platform enabled the rapid synthesis of >1000 PhotoAffinity Bits (HTC-PhABits) in 384-well plates in 24 h and their subsequent screening as crude reaction products with a protein target without purification. Screening the HTC-PhABit library with carbonic anhydrase I (CAI) afforded 7 hits (0.7% hit rate), which were found to covalently crosslink in the Zn^2+^ binding pocket. A powerful advantage of the D2B-HTC screening platform is the ability to rapidly perform iterative design–make–test cycles, accelerating the development and optimisation of chemical tools and medicinal chemistry starting points with little investment of resource.

## Abstract 2

A photoreactive fragment screening platform employing direct-to-biology high-throughput chemistry (D2B-HTC) for the rapid iterative synthesis and screening of libraries of photoaffinity bits.

## Graphical abstract

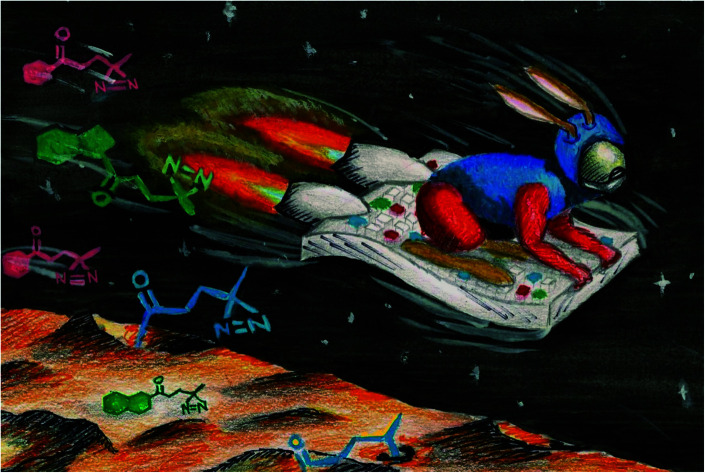

## Introduction

Human genetics and functional genomic studies are providing insights into potential therapeutic targets on an unprecedented scale.^1–5^ Validation of these targets in early-stage discovery relies on the rapid identification of tool molecules that can inform on the mechanism of action best suited to therapeutic intervention.^6^ Reactive fragment-based technologies have recently emerged as a powerful approach for the identification of tool molecules.^7–12^ Reactive fragments leverage the ability of relatively small compounds (<300 Da) to efficiently explore chemical space, coupled to a reactive functionality to enable the capture and robust detection of transient fragment-protein interactions.^13–18^ Crucially, covalent bond formation opens up a suite of follow-up studies, such as site identification and cellular target engagement, providing valuable tools for the study of emerging targets.^19–21^

**Fig. 1:**
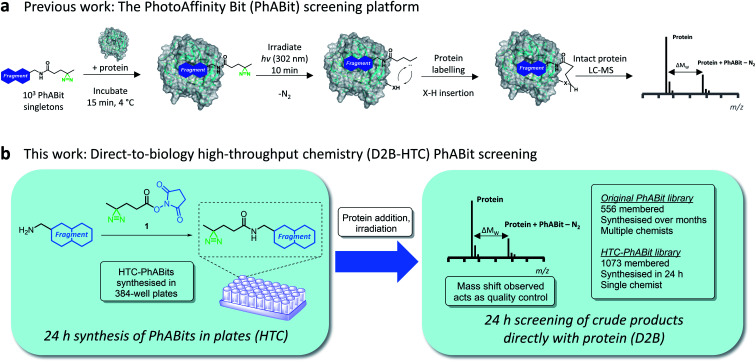
(a) Schematic representation of previous work describing the PhotoAffinity Bit (PhABit) screening technology. (b) D2B-HTC PhABit screening platform. PhABits can be synthesised in 384-well plates in 24 h and screened as crude reaction products with a protein target.

Initial reports of reactive fragments have focussed on the use of cysteine-reactive electrophilic fragment libraries (*e.g.* α,β-unsaturated carboxylic esters and amides).^7–12,17,54^ These platforms have enabled the development of numerous chemical probes, including for previously unliganded proteins.^8,9^ A limitation of this approach is the requirement for a cysteine residue in the proximity of the binding pocket. More recently, platforms have been developed employing photoreactive groups.^22–26^ Irradiation by UV light converts the photo-labile functionality into a highly reactive intermediate (typically a carbene/nitrene) that covalently inserts into a proximal amino acid residue.^27–29,56^ This expands the number of protein pockets amenable to reactive fragment screening, greatly enhancing the applicability of such strategies. This approach was employed by Parker, Cravatt and co-workers to enable the mapping of fragment-protein interactions in cells using fully functionalised fragments (FFFs).^30,31^ Building upon this technology, we recently reported a photoreactive fragment screening platform (PhABits) which enabled the rapid identification of tools for purified proteins of interest to support early-stage biomedical research (Fig. 1a).^32^

A key challenge for reactive fragment platforms, and indeed any chemical tool/medicinal chemistry campaign, is the optimisation of initial hits to generate more potent and selective tool compounds.^14,55^ In these early medicinal chemistry design–make–test cycles, compound synthesis presents the major bottleneck. We anticipated an opportunity to accelerate hit optimisation through the combination of high-throughput chemistry (HTC) with direct-to-biology (D2B) screening. The approach would involve the synthesis of whole libraries of reactive fragments in a 384-well plate-based format followed by direct screening of the crude products with a protein target.^33–39^ Crucially, the intact protein LC-MS screening method provides a quality control for the chemical identity of any hit fragments, as determined by the observed protein mass shift. Thus reactive fragment screening would overcome the challenges encountered in historical D2B-HTC approaches, where the prevalence of false positives due to low levels of impurities in the reaction mixtures has prohibited widespread adoption.^40–42^

Herein, we report a D2B-HTC photoreactive fragment screening platform (HTC-PhABits). A library of >1000 HTC-PhABits were synthesised in 384-well plates and screened directly without purification by irradiation in the presence of a purified protein of interest (Fig. 1b). An LC-MS analytical method allowed robust identification of light-induced covalent crosslinking events, leading to expedient identification of fragment hits. Crucially, HTC was subsequently used to synthesise and screen a second-generation library of 100 hit analogues to improve hit rate and crosslinking yield. HTC-PhABits thus offer a significant advance on our previously reported 556-membered PhABit library, where synthesis and purification were conducted over a number of months and hit optimisation was hindered by the resource requirements of hit analogue synthesis. We expect this platform to substantially accelerate PhABit synthesis and screening and enable rapid design–make–test cycles with minimal investment of resource and in short timeframes, thereby avoiding the compound synthesis bottleneck. Coupled with informative hit follow-up studies, such as binding site identification, we anticipate the platform will be widely applicable for identification of tool molecules for emerging protein targets of interest.

## Results and discussion

### Development of a high-throughput PhABit synthesis

**Fig. 2:**
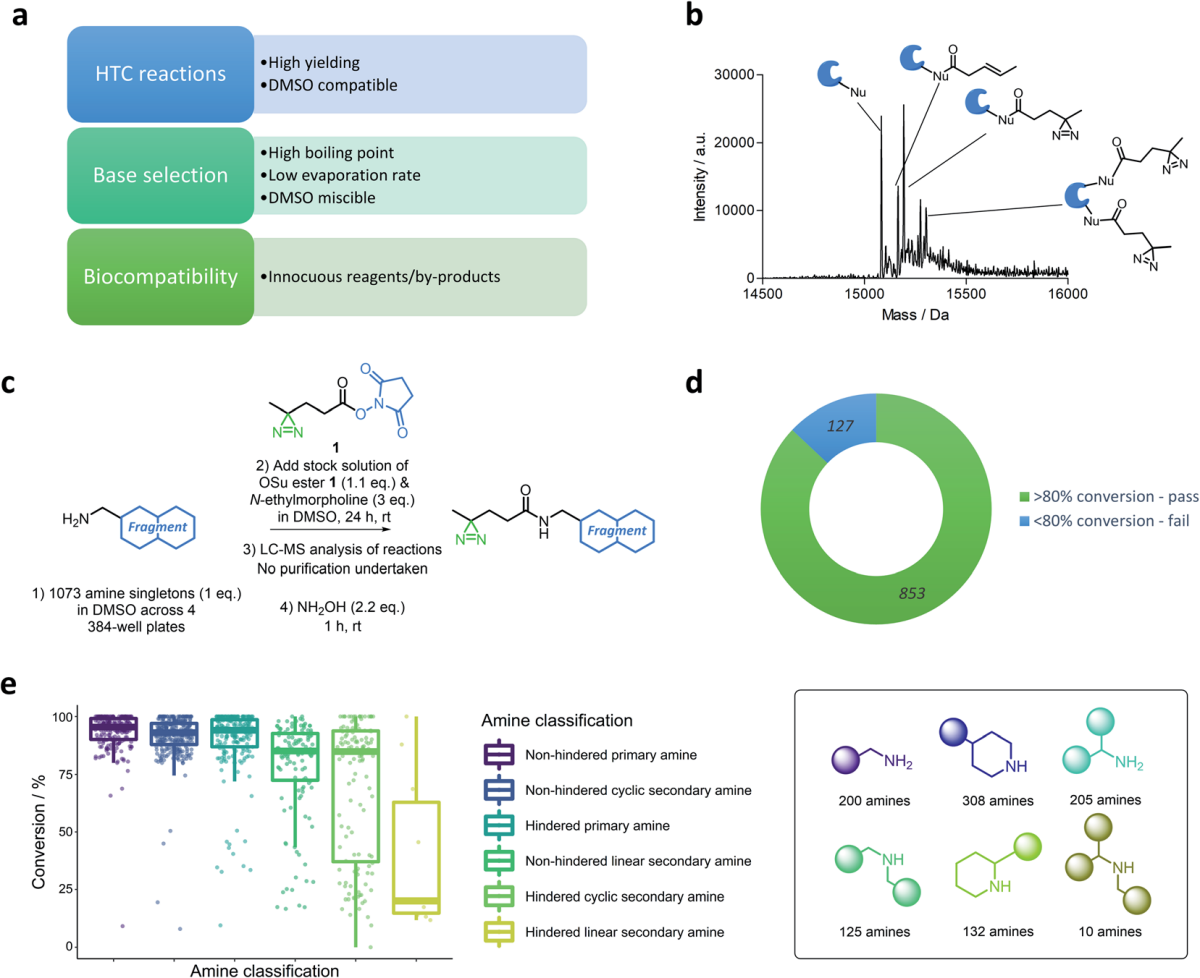
(a) Criteria required for the development of a high-throughput chemistry direct-to-biology workflow. (b) Mass spectrum displaying the non-specific adducts formed between an exemplar protein (BRD4-BD1) and an irradiated (302 nm, 10 min) HTC-PhABit containing unreacted succinimide activated ester **1**. (c) Protocol for the in-plate synthesis of a library of high-throughput PhABits (HTC-PhABits). 1073 PhABits were synthesised without purification across 4 × 384-well plates. (d) Success rate of the HTC-PhABit synthetic protocol, omitting the 54 impure amine starting materials and the 39 reactions for which conversion was unable to be calculated. (e) Boxplot for the conversion of the HTC-PhABit reactions grouped by the classification of the amine. Amines were classified according to the following categories: hindered *vs.* unhindered, secondary *vs.* primary and linear *vs.* cyclic.

A D2B-HTC approach to reactive fragment screening could provide an efficient means to grow and optimise reactive fragment libraries. Such a platform requires robust, high yielding, DMSO-compatible reactions, obviating the need for purification. Furthermore, the reactions should be biocompatible with the protein screening step and form innocuous by-products (Fig. 2a).

The succinimide-activated (OSu) amide coupling provided a suitable option, as a high yielding reaction that affords *N*-hydroxysuccinimide as the only by-product.^43^ It was anticipated that the diazirine OSu-ester **1** could be added to a plate containing fragment amines to afford the desired HTC-PhABits in sufficient purity to allow direct screening against targets of interest. Diazirine **1** was selected as the photoreactive functionality based on the high reactivity of the associated carbene, which limits non-specific labelling.^24,28,44^

Reaction conditions were optimised to enable coupling of the diazirine OSu-ester **1** with a library of amines in 384-well plate format at room temperature. DMSO was selected as the reaction solvent to ensure direct compatibility with biochemical assays. The selection of base for the reaction was governed by both miscibility with DMSO and boiling point, as evaporation rates can be a concern in plate-based syntheses (Fig. 2a).^45^ Common bases, triethylamine and *N*,*N*-diisopropylethylamine, were found to be unsuitable for the reaction as they were immiscible with DMSO. *N*-Methylmorpholine (NMM) exhibited good miscibility, however, was found to evaporate rapidly from DMSO in a 384-well plate format (b.p. = 116 °C, half-life = 150 min, recorded at ambient temperature) (Fig. S1†). Therefore, *N*-ethylmorpholine (NEM) was employed, which retained good miscibility with DMSO and exhibited a slower evaporation rate (b.p. = 138 °C, half-life = 270 min, recorded at ambient temperature) (Fig. S1†). Trial reactions were performed on a selection of 4 representative amines (1 eq. amine (**S1–4**), 1.1 eq. **1**, 3 eq. NEM, 100 mM in DMSO, total volume = 80 μL). Alkyl amines *p*-methoxybenzylamine (**S1**) and *p*-nitrobenzylamine (**S2**) afforded complete conversion to the desired product after 24 hours at room temperature, while aniline (**S3**) and *N*-methylaniline (**S4**) displayed poor conversion (Table S1†). Aryl amines were therefore excluded from this library of amine fragments.

Initial studies on the biocompatibility of these crude reaction products with protein screening indicated that unreacted succinimide ester **1** could react non-specifically with nucleophiles on the protein surface forming multiple adducts, confounding the mass spectrometry analysis (Fig. 2b). Therefore, a quenching step was introduced to remove unreacted OSu-ester **1** by reaction with hydroxylamine (NH_2_OH_(aq)_, 2.2 eq., 1 h, rt).

### Library synthesis

With the D2B-HTC reaction conditions optimised (Fig. 2c), a library of 1073 alkyl-amine fragments (mean *M*_w_ 211 Da) were selected from the GSK compound collection using clustering on molecular fingerprints (ECFP4) to maximise diversity (for library properties see Fig. S2†). This library included 288 alkyl-amines present in our previously reported PhABit library.^32^ The amine fragments were plated across 4× 384-well plates (10 mM DMSO) and subjected to LC-MS analysis to assess initial purity. This resulted in 54 amines being removed from subsequent analysis due to insufficient purity. To each well was added a stock solution of compound **1** (1.1 eq.) and *N*-ethylmorpholine (3 eq.) in DMSO (Fig. 2c, final HTC-PhABit concentration 5 or 8 mM). The plates were sealed and incubated at room temperature for 24 hours. The wells were then analysed directly by LC-MS to assess conversion. The success of the reaction was determined by purity (>80% purity was classified as a pass (626 reactions)). HTC-PhABits with purity <80% were further investigated to determine the cause of the low LC-MS purity according to the flow diagram provided in the ESI (Fig. S3†). This more in depth analysis rescued 227 reactions, to afford a total of 853 successful reactions. The conversion was unable to be determined for 39 reactions due to either failed LC-MS or the product not being visible by LC-MS. Therefore, conversion was calculated for a total of 980 reactions. The HTC reaction success rate was therefore 87% when considering only amines for which a conversion figure could be determined and omitting those with impure amine starting materials (Fig. 2d, for full breakdown see Fig. S4†).

To investigate the influence of amine structure on the success rate of the synthetic procedure, the amines were grouped into 6 categories according to: primary/secondary, cyclic/linear and hindered/unhindered (amines with ≥2 substituents α to the nitrogen were classified as hindered) (Fig. 2e). Primary amines, both hindered and unhindered, afforded high levels of conversion with few failures (median conversion = 94 ± 14% (*n* = 205) and 95 ± 9% (*n* = 200) respectively). Unhindered cyclic/linear secondary amines also afforded good conversions (median conversion = 93 ± 9% (*n* = 308) and 85 ± 21% (*n* = 125) respectively), while cyclic/linear secondary amines featuring an α-substitution gave the largest variation in conversion and a significant number of failures (median conversion = 85 ± 30% (*n* = 132) and 20 ± 34% (*n* = 10) respectively). The low conversions of hindered secondary amines can be rationalised by sterics, while the lower conversions observed with linear *versus* cyclic secondary amines can be attributed to steric and conformational factors. It is possible that these amines could be rescued through the use of alternative coupling conditions or elevated temperature.

### Direct-to-biology HTC-PhABit screen against human carbonic anhydrase I

Human carbonic anhydrase I (CAI) was selected for validation of the HTC-PhABit library as a proof of concept on account of the availability of reported crystallographic information and known inhibitors for validation studies. CAI is a member of a family of Zn^2+^ binding metalloenzymes that catalyse the physiological conversion of CO_2_ to HCO_3_^−^, a key respiratory process and method of pH regulation.^46,47^ A number of inhibitors of carbonic anhydrase isozymes have been reported, which commonly feature a primary sulfonamide group.^46,47^ Carbonic anhydrase inhibitors have been used to treat glaucoma, oedema, cancer and can act as diuretics.^46^

**Fig. 3:**
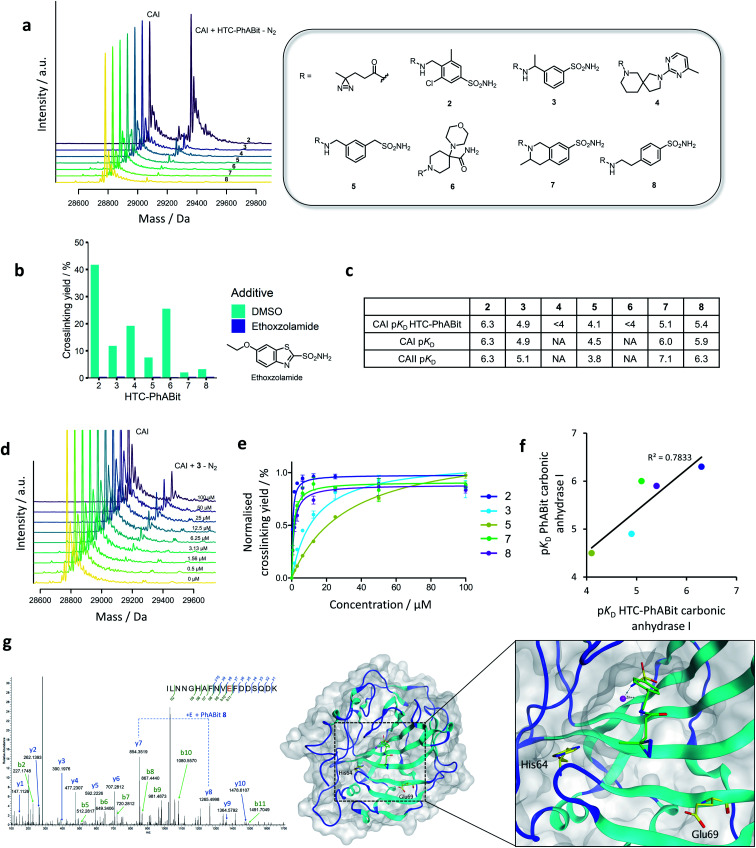
(a) Summary of the single shot screen of the HTC-PhABit library against carbonic anhydrase I (CAI), showing the structures and corresponding mass spectra for 7 hits. (b) Crosslinking competition study with known CA inhibitor, ethoxzolamide. (c) p*K*_D_ values of the five sulfonamide containing PhABits as both crude products and purified with carbonic anhydrase I & II (concentration-response data recorded in triplicate). (d) Exemplar concentration-response mass spectra for PhABit **3**. (e) Normalised concentration-response curves for the five purified sulfonamide containing PhABits with carbonic anhydrase I. (f) Comparison of the p*K*_D_ values obtained through the concentration-response studies of the five sulfonamide containing hits with CAI as both crude products and purified compounds. (g) Left: LC-MS/MS spectra of the peptide _59_ILNNGHAFNVEDDSQDK_76_ crosslinked to **8** indicating Glu69 as one of the sites of crosslinking; Right: the X-ray crystal structure of carbonic anhydrase II (PDB: 3CAJ) virtually docked with PhABit **8**, highlighted are the two residues that were modified by all PhABits (His64 and Glu69).

CAI (1 μM, PBS) was incubated with the HTC-PhABit library (100 μM) in 384-well plates before irradiation with UV light (10 min, 302 nm). The HTC-PhABit library was used assuming quantitative conversion during library synthesis. Following irradiation, the plates were directly analysed by intact protein LC-MS to identify any wells where photo-induced crosslinking had occurred. The majority of wells gave spectra that contained a single peak corresponding to unmodified protein, which provided validation of the biocompatibility of the HTC protocol. Hit fragments were characterised by those that displayed >1.5% crosslinking yield. Such hits contained a second peak at a mass shift corresponding to the mass of [PhABit-N_2_], which enabled identification of these as binding hits and also provided confirmation of the chemical identity of the hit. It is important to note that the absolute value of the crosslinking yield is not indicative of binding affinity, but a composite of the binding affinity and the crosslinking efficiency of the carbene intermediate generated upon irradiation. Thus, crosslinking yields were not used to rank PhABit affinity. Of the 1073 HTC-PhABits screened, 7 displayed crosslinking yields >1.5%, corresponding to a 0.7% hit rate (Fig. 3a, S5 and Table S2†). Many of these hits contained aryl primary sulfonamide functionalities (**2**, **3**, **5**, **7** and **8**, Fig. 3a) which are known to coordinate to the Zn^2+^ ion in the active site and therefore set up reversible binding prior to covalent crosslinking, while some novel chemotypes were also observed (**4** and **6**, Fig. 3a).^46^

### Concentration response and competition assay

Hit follow-up was performed to further validate the detected HTC-PhABit hits and to subsequently compare the behaviour of hits derived from D2B-HTC *versus* traditional synthesis and purification. Competition studies with ethoxzolamide (*K*_I_ 25 nM) were carried out to elucidate whether the 7 HTC-PhABit hits were binding in the same binding site.^46^ The 7 unpurified HTC-PhABits (100 μM) were incubated and irradiated in the presence of either ethoxzolamide (100 μM) or DMSO as a control. All 7 HTC-PhABits (**2–8**), including novel structures **4** and **6**, were displaced by ethoxzolamide, suggesting these HTC-PhABits were binding in the Zn^2+^ pocket (Fig. 3b and Table S3†).

To determine the binding affinity of the 7 HTC-PhABit hits, the unpurified hits were incubated at varying concentrations (0–100 μM) with CAI protein (1 μM, PBS) and irradiated (302 nm, 10 min). Crosslinking yields, as determined by intact protein LC-MS analysis, were plotted *vs.* concentration (Fig. S6†). Only 5 of the 7 HTC-PhABits screened in concentration-response displayed a measurable dissociation constant (p*K*_D_ = 4.1–6.3, Fig. 3c). These 5 HTC-PhABits (**2**, **3**, **5**, **7** and **8**) all contained a primary sulfonamide. The strong binding affinities for **2**, **7**, and **8** are consistent with *K*_I_ values of known fragment-like sulfonamides. For example, ethoxzolamide, acetazolamide and methazolamide (*M*_w_ < 300 Da) have reported *K*_I_ values in the range 25–250 nM.^46^ HTC-PhABits **4** and **6** showed a linear relationship between concentration and crosslinking yield, suggesting a *K*_D_ value >100 μM (Fig. S6†).

To investigate the consequence of screening with HTC-generated crude products, the concentration-response study was repeated following re-synthesis and purification of the 5 sulfonamide hits (**2**, **3**, **5**, **7** and **8**) (Fig. 3c–f). There was good correlation between the p*K*_D_ values obtained for HTC-PhABits and the purified PhABits (Fig. 3f, *R*^2^ = 0.78). It was noted that the HTC-PhABit study underestimated the binding affinity in 3/5 cases, likely due to lower purity. However, the overall correlation provides support for the utility of the D2B-HTC protocol in rapidly characterising hits, prior to any subsequent re-synthesis of compounds of interest.

The crosslinking yields of PhABits **2**, **3**, **5**, **7** and **8** with carbonic anhydrase II (CAII) were investigated to explore isoform selectivity (Table S4†). All PhABits were found to crosslink to this related isoform, and subsequent concentration-response studies differentiated between ‘pan-CAI/II’ fragments, and those that offered selectivity for a single isoform (Fig. 3c and S8†).

### Site of crosslinking identification

A key advantage of covalent crosslinking is the ability to determine the site of binding by leveraging LC-MS/MS analysis. CAII labelled with a PhABit (**3**, **5**, **7** and **8**) was subjected to digestion with trypsin, followed by LC-MS/MS analysis. CAII was selected for this study due to higher levels of crosslinking observed for the hit PhABits. For all four compounds, a data-dependent analysis identified peptide 59–76 ILNNGHAFNVEDDSQDK as carrying the modification (282.1038 Da for **3**, **5**, **8** and 308.1195 Da for **7**). No modified peptides were observed in the non-irradiated controls. To determine the specific residue of modification, the peptide digests were subjected to parallel reaction monitoring (PRM) LC-MS/MS, targeting the [M + 3H]^3+^ ion of the 59–76 modified peptide. Manual interpretation of the PRM MS/MS spectra identified His64 and Glu69 as major sites of modification for the four hit compounds (for example spectra see Fig. 3g).

The residues His64 and Glu69 are located at the entrance of the Zn^2+^ binding pocket. A virtual docking of PhABit **8**, based on the reported binding mode of ethoxzolamide (PDB: 3CAJ), highlighted the two residues are suitably positioned for crosslinking to the docked PhABit (Fig. 3g).^48^ Interestingly, the tetrapeptide _65_AFNV_68_ between the two modified residues was largely unmodified by the PhABits (only PhABit **7** labelled Ala65 in very low stoichiometry) (Fig. S9†). The lack of labelling observed for these four non-polar amino acids highlights a potential selectivity of the activated diazirine towards particular residues. This is consistent with previous reports that carbenes more frequently label polar residues.^49^ Semi-quantitative analysis of relative crosslinking yields indicated that all PhABits displayed highest labelling at Glu69, with the exception of PhABit **5** which displayed a higher labelling of His64.

### Iterative library synthesis using direct-to-biology high-throughput chemistry

A key advantage of D2B-HTC is the ability to synthesise iterative libraries of hit-analogues in short timeframes to identify more potent binders, as well as improve target selectivity through off-target screening. It is anticipated that early stage discovery could be supplemented by rapid D2B-HTC design–make–test cycles to generate large volumes of data for protein targets, enabling rapid exploration of SAR and evolution towards optimised tools.

**Fig. 4:**
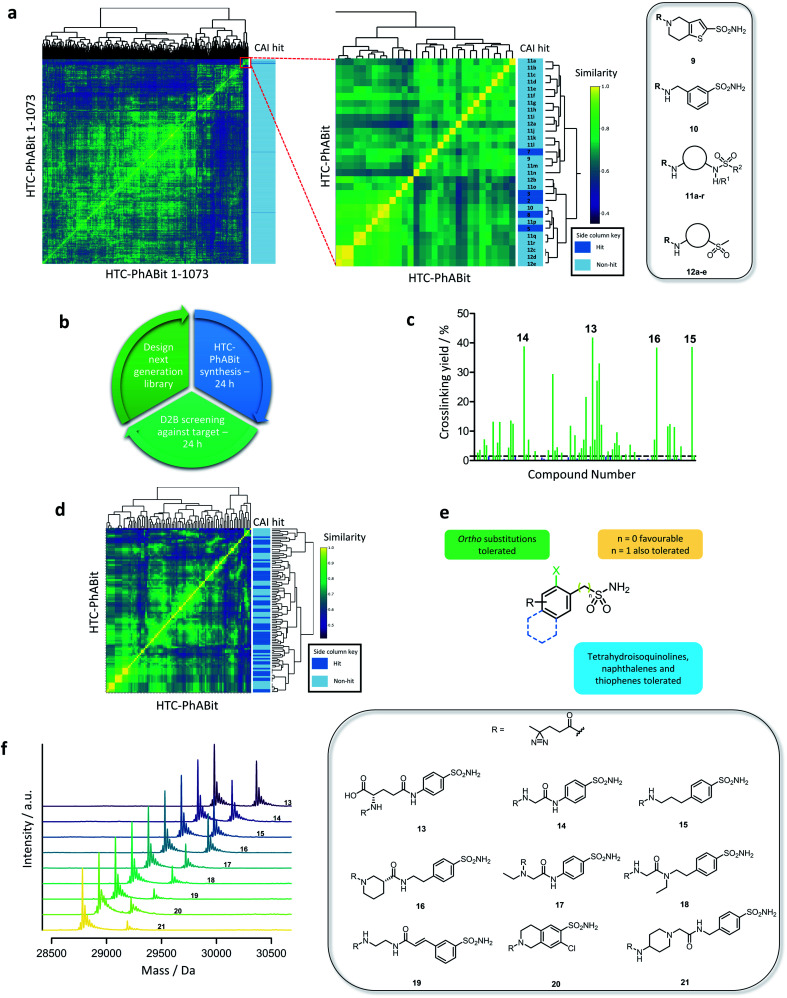
(a) Visualisation of the library through similarity matrix and heatmap generation with a side column indicating crosslinking to CAI (dark blue: hit) along with structures of non-crosslinking primary sulfonamides (**9–10**) and generalised structures of non-crosslinking secondary/tertiary sulfonamides (**11a–r**) and sulfones (**12a–e**). Dendrograms link structurally similar compounds and are a result of the hierarchical clustering method used. (b) Proposed iterative design–make–test hit ID protocol utilising the D2B HTC-PhABit platform to identify binders. (c) Crosslinking yields of the 100 HTC-PhABits with CAI (green: >1.5% crosslinking, blue: <1.5% crosslinking). (d) Heatmap displaying the similarity between the members of the 100-membered second-generation HTC-PhABit library with a side column indicating crosslinking to CAI (dark blue: hit). (e) Generalised structure of a hit primary sulfonamide summarising the tolerated features for binding in the Zn^2+^ binding pocket. (f) Structures and corresponding mass spectra for the 9 highest crosslinking hits from the single shot screen.

Interpretation of the initial CAI screen and elucidation of any structure activity relationships (SAR) emerging was required for the design and selection of a second-generation HTC-PhABit library. Therefore, a similarity matrix of the HTC-PhABit library was constructed and visualised as a heatmap. Using software package RCDK in R-Studio, the library of 1073 HTC-PhABits were converted to molecular fingerprints (MACCS) and the similarity (Tanimoto) was calculated between all library members.^50–53^ Hierarchical clustering based on structural features afforded a 1073 × 1073 similarity matrix, which was visualised as a heatmap (Fig. 4a). A region of close similarity (inset Fig. 4a, S10 and Table S5†) was identified that contained 7 primary sulfonamides, including hits (**2**, **3**, **5**, **7** and **8**) and non-hits (**9** and **10**, Fig. 4a), as well as several secondary/tertiary sulfonamides (**11a–r**) and aryl sulfones (**12a–e**) each of which did not crosslink to the protein in the initial screen. Upon closer inspection, compound **9** showed minor crosslinking (1.0%), which did not meet the threshold to be classified as a hit (>1.5%). Compound **10**, a close analogue of hit **3**, showed no crosslinking, suggesting the benzylic methyl group was either crucial to potency or generated a conformation favourable to crosslinking.

Examination of the structures of the other HTC-PhABits within this region of the heatmap provided further insight into the SAR around the hits. Numerous secondary/tertiary sulfonamides (**11a–r**) and aryl sulfones (**12a–e**) were present that did not display crosslinking to CAI (Fig. 4a), highlighting the primary sulfonamide as a privileged chemotype for carbonic anhydrase, a feature consistent with previously reported inhibitors.^46^

Taking this information together, a 100-member library of hit-analogues was designed based on the structures of initial hits **2**, **3**, **5**, **7** and **8** comprising 83 primary sulfonamides as well as 17 secondary/tertiary sulfonamides and sulfones as negative controls (Fig. 4b) (for library properties see Fig. S11†). The library was synthesised and screened against CAI following the established D2B-HTC protocol, which yielded a significant improvement in hit rate (52 hits >1.5% crosslinking) (Fig. 4c). The crosslinking yields were also improved with 17 fragments giving greater than 10% labelling. This is particularly impactful to the PhABit platform, in providing a means to overcome the challenges associated with the often low crosslinking yields of photoreactive fragments. D2B-HTC cycles can thus enable optimisation toward PhABits that give high crosslinking yields, allowing them to serve as valuable tools for the study of proteins of interest.

Inspection of the hit and non-hit fragment structures provided further insights into the SAR within the Zn^2+^ binding pocket (Fig. 4d–f). All 52 hits contained a primary sulfonamide, while none of the secondary/tertiary sulfonamides and sulfones displayed crosslinking. The majority of the hits (77%) featured a primary sulfonamide directly bonded to a phenyl ring. Other ring systems were also tolerated such as tetrahydroisoquinoline sulfonamide (7 hits), thiophene sulfonamide (1 hit), benzyl sulfonamide (1 hit) and naphthalene sulfonamide (1 hit). Several hits (19%) featured an *ortho* substituent, suggesting additional space in the pocket adjacent to the Zn^2+^ ion. Conversely, inspection of the non-hits identified 1*H*-indole-5-sulfonamide, 1*H*-indole-5-benzylsulfonamide and 1*H*-isoindoline-5-sulfonamide as potentially non-binding primary sulfonamides. However, a limitation of analysing negative hits from photoaffinity labelling experiments is the potential for false negatives due to poor crosslinking.

Overall, the application of iterative design–make–test cycles to the D2B-HTC-PhABit platform enabled the identification of a large number of new elaborated hits. Furthermore, a higher proportion of these hits displayed >10% crosslinking making them suitable chemical tools for downstream labelling experiments. It has also been demonstrated that detailed SAR patterns can be elucidated by analysing the results from the iterative screening process.

## Conclusion

The D2B-HTC platform developed here provides a powerful approach to enable the rapid iterative synthesis of large libraries (10^3^) of photoreactive fragments and subsequent screening with single purified protein targets of interest directly as crude reaction products, without any purification. Such a screening platform is anticipated to enable the facile identification and optimisation of hit fragments into more potent and selective tool molecules, overcoming the significant synthetic bottleneck in previous reactive fragment screening platforms. The HTC-PhABit approach exploits the intrinsic quality control of the mass spectrometric read-out, confirming the identity of hit fragments and thereby overcoming the challenges associated with false positives, which have hindered D2B-HTC platforms to date.

The HTC-PhABit approach enabled the rapid generation of >1000 PhABits that were directly screened against carbonic anhydrase I. The screening process identified multiple micromolar hits, which were validated by re-synthesis and purification. Follow-up studies enabled determination of potency and site of binding. In a second screening cycle, a library of 100 hit analogues was rapidly synthesised and screened to yield 52 additional hits with improved crosslinking yields. This follow-up screen provided multiple insights into the SAR with very little investment of resource relative to traditional medicinal chemistry design–make–test cycles. With the rate and scale of data generation offered by D2B-HTC, we envision that this platform will provide an opportunity to rapidly identify more potent and selective tool molecules for emerging protein targets. In order to fully realise the potential of this approach, a key next step will be to expand the scope of D2B-HTC-compatible reactions, providing multiple avenues for the development of high-quality tools.

## Author contributions

RPT, REH, SB and FZ performed experiments and analysed data. RPT, EKG, JTB and NCOT drafted the paper. PP carried out computational studies. JTB, NCOT, MMH and DH contributed to experiment design. DJF developed code for data visualisation.

## Conflicts of interest

There are no conflicts to declare.

## Supplementary Material

SC-012-D1SC03551G-s001
