# Supplementary material for: A direct-to-biology high-throughput chemistry approach to reactive fragment screening
Source: Chem Sci. 2021 Aug 11;12(36):12098–106. doi: 10.1039/d1sc03551g (PMC8457371; doi:10.1039/d1sc03551g)
Supplement: SC-012-D1SC03551G-s001 [file SC-012-D1SC03551G-s001.pdf]

Electronic Supplementary Material (ESI) for Chemical Science.
This journal is © The Royal Society of Chemistry 2021

A direct-to-biology high-throughput chemistry approach to reactive fragment screening

Ross P. Thomas, Rachel E. Heap, Francesca Zappacosta, Emma K. Grant, Peter Pogány, Stephen Besley, David J. Fallon, Michael M. Hann, David House, Nicholas C. O. Tomkinson and Jacob T. Bush*

Supplementary information

Contents

1 . Supplementary Figures 4
2. General Methods 18
2.1 Solvents, reagents and consumables 18
2.2 Irradiation 18
2.3 Nuclear magnetic resonance (NMR) spectroscopy 18
2.4.1 Liquid chromatography-mass spectrometry (LC-MS) for small molecules 19
2.4.2 LC-MS of HTC-PhABit library 20
2.5 Mass directed automated preparative HPLC (MDAP) 20
2.6 Infrared Spectroscopy 21
2.7 Melting Point 21
2.8 High-resolution mass spectrometry 21
2.9 TLC and Flash column chromatography 22
2.10 Intact Protein LC-MS 22
2.11 Centrifuge 24
2.12 Molecular docking 25
3. Library synthesis 25
3.1 Amine selection 25
3.2.1 HTC-PhABits group A (final concentration 5 mM) 26
3.2.2 HTC-PhABits group B (final concentration 8 mM) 26
4. Screening the HTC-PhABit library 27
4.1 HTC-PhABit plate preparation 27
4.2 Protein addition and irradiation protocol 27
4.3 Single shot screen of 1073 HTC-PhABits with CAI 27
5. Follow-up studies: competition, concentration-response, LC-MS/MS 29
5.1 Competition studies with ethoxzolamide 29
5.2 Concentration response procedures 29
5.2.1 Concentration-response with HTC-PhABits 29
5.2.2 Concentration-response with purified PhABits 31
5.3 Site of crosslinking LC-MS/MS studies 32
6. Iterative HTC-PhABit library synthesis and screening 34
6.1 Amine selection 34
6.2 Second generation HTC-PhABit library 34
6.3 Screening the second-generation HTC-PhABit library with CAI 35

7. Compounds 37
7.1 Synthesis of covalent warhead 37
7.2 General procedure for the synthesis of primary sulfonamide hits 39
7.3 Primary sulfonamide hits 40
8. References 43

1 . Supplementary Figures

a

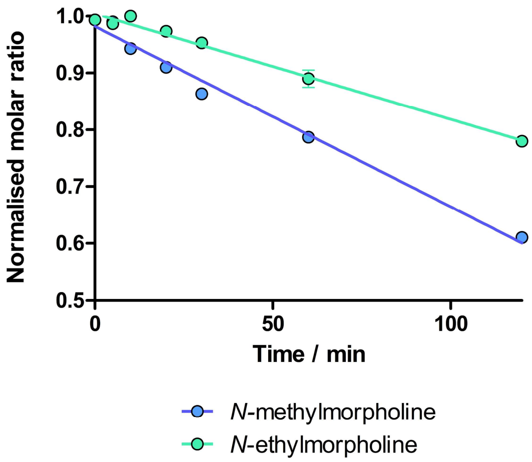

b

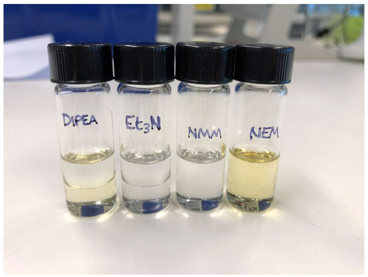

c

**Base**	**Boiling point[a] / °C**	**Miscible with DMSO**	**Half-life in 384-well plate in DMSO / min**
DIPEA	127	N	NA
Et_3N	89	N	NA
NMM	116	Y	150
NEM	138	Y	270

Figure S1a) Evaporation rate of N-methylmorpholine (NMM) and N-ethylmorpholine (NEM) from a Greiner 384-well plate in DMSO, recorded at ambient temperature. b) Miscibility of the four selected based with DMSO, 1 mL of base was combined with 1 mL DMSO. c) Summary of the chemical properties of the four bases including half-lives of N-methylmorpholine and N-ethylmorpholine in a 384-well plate in DMSO. [a]Data taken from PubChem.

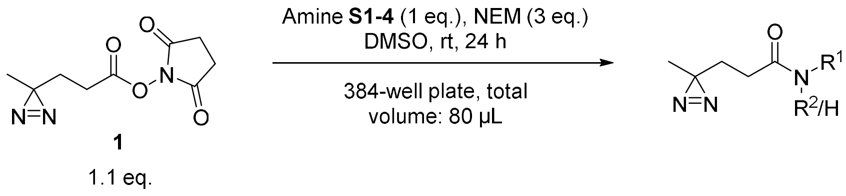

Substrate	Conversion by LC-MS after 1 h / %	Conversion by LC-MS after 24 h / %	Conversion by ^1H NMR after 1 h / %	Conversion by ^1H NMR after 24 h / %
p-Methoxybenzylamine (**S1**)	90	91	100	100
p-Nitrobenzylamine (**S2**)	99	98	100	100
Aniline (**S3**)	2	13	0	9
N-methylaniline (**S4**)	0	0	0	6

Table S1. Test reactions of four representative amines with the selected conditions (1 eq. amine, 1.1 eq. ***1****, 3 eq. NEM, DMSO, 24 h, rt). Success measured by conversion by ^1H NMR and LC-MS of the crude reaction mixtures. Values quoted represent the mean across three replicates.*

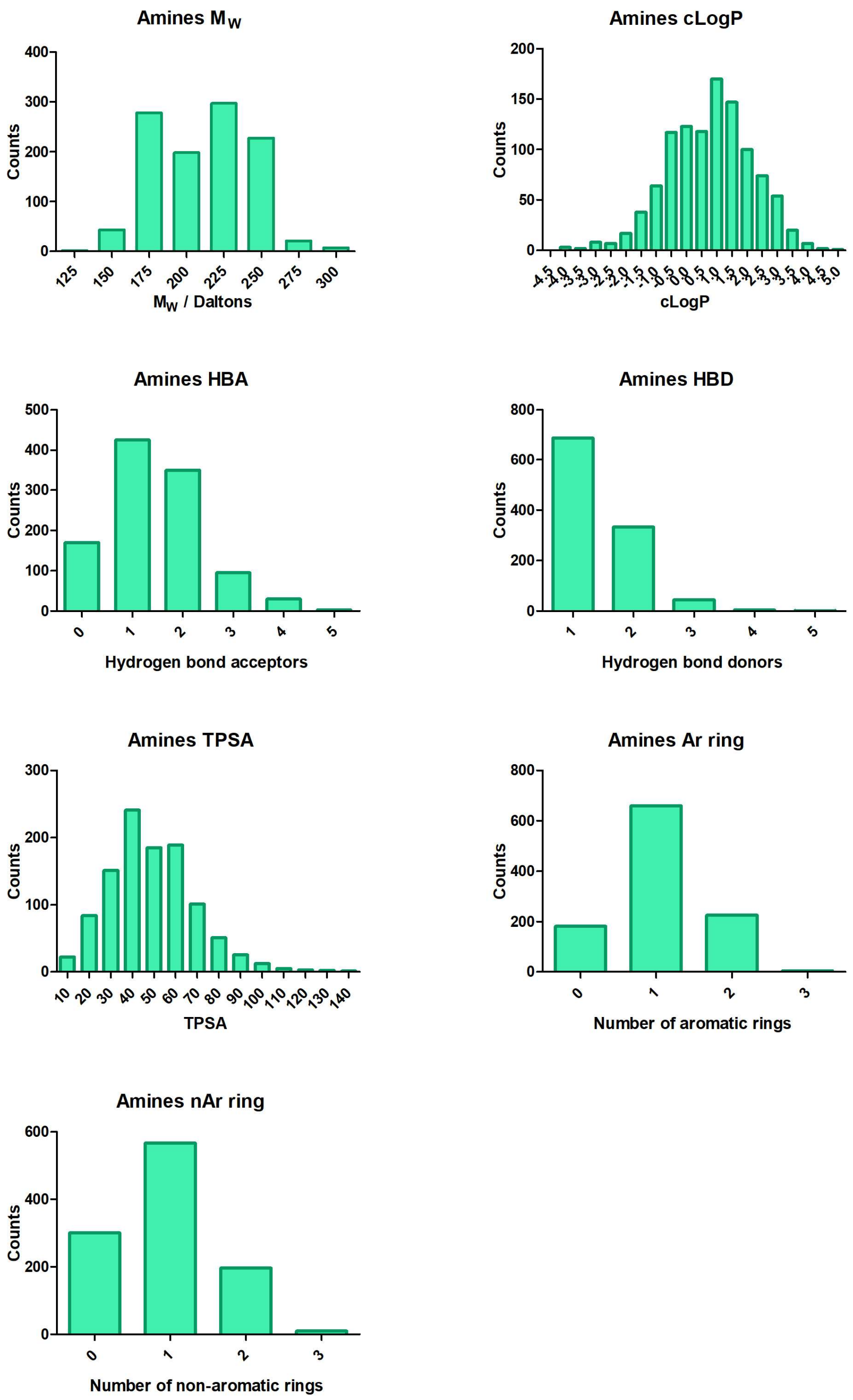

Figure S2. Binned properties of the 1073 membered amine fragment library. Free 'uncapped' amines were subjected to the analysis.

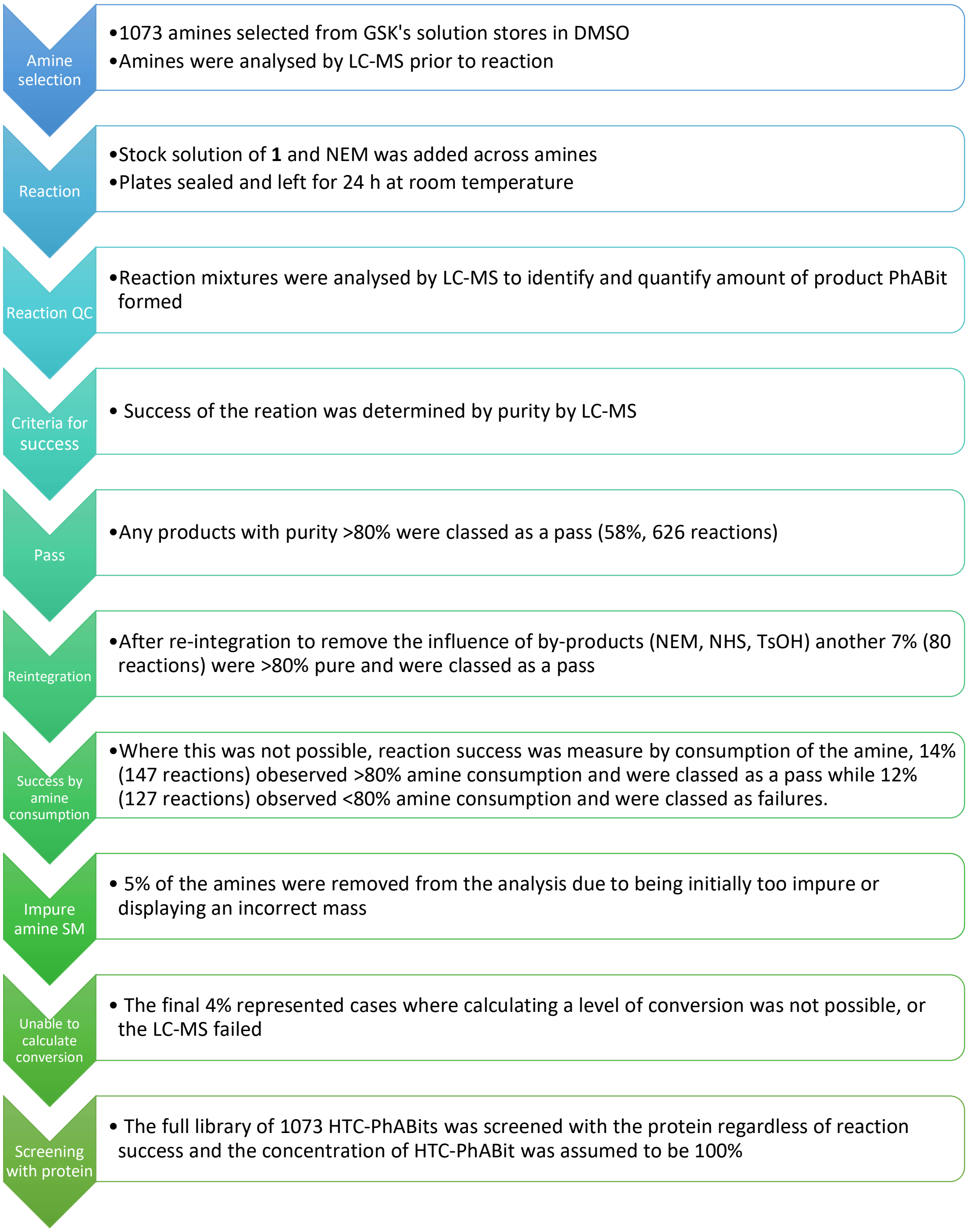

Figure S3. Flow diagram showing how the success of the HTC reactions was assessed. The conversion was measured by LC-MS analysis directly from the plates.

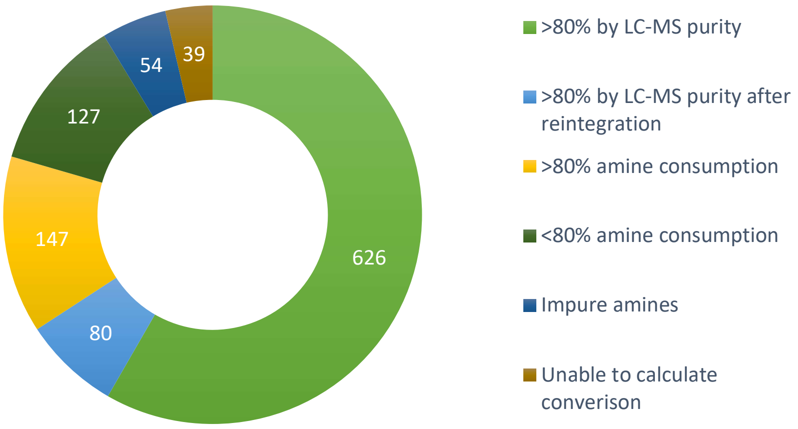

Figure S4. Pie chart to show breakdown of the successes and failures of the HTC-PhABit synthesis protocol.

HTC-PhABit	Crosslinking yield / %
2	51.1
3	10.3
4	7.4
5	4.9
6	2.4
7	2.0
8	2.5

Table S2. Crosslinking yields and well positions of the 7 HTC-PhABits that displayed crosslinking to carbonic anhydrase I.

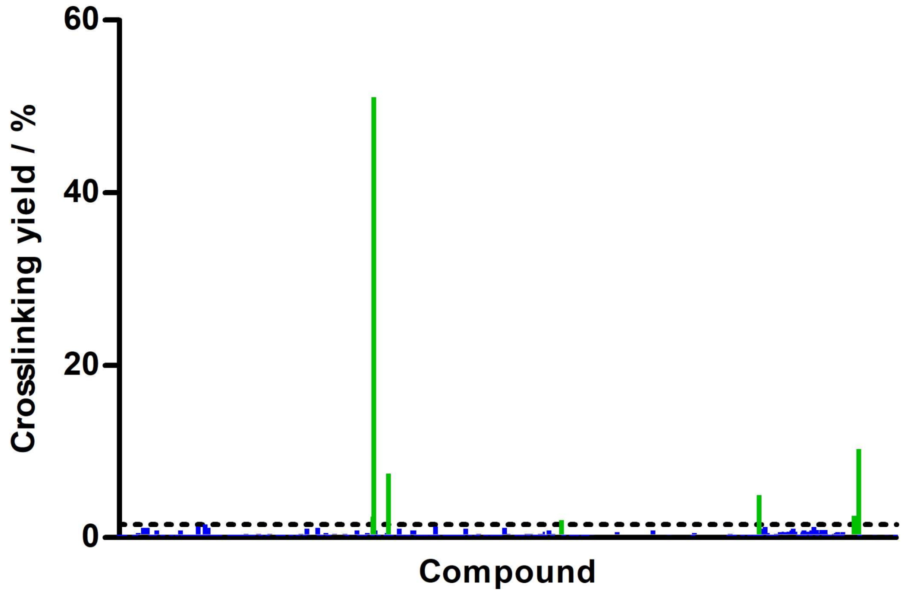

Figure S5. Crosslinking yield for each compound of the 1073 membered HTC-PhABit library. Compounds with >1.5% crosslinking are depicted as green bars, all others are in blue.

Table S3. Crosslinking yields of the hit HTC-PhABits in the presence of ethoxzolamide and DMSO as a control.

HTC-PhABit	Crosslinking yield +DMSO	Crosslinking yield +ethoxzolamide
2	41.7	0.4
3	11.8	0.5
4	19.2	0.1
5	7.5	0.4
6	25.5	0.3
7	2.0	0.4
8	3.2	0.4

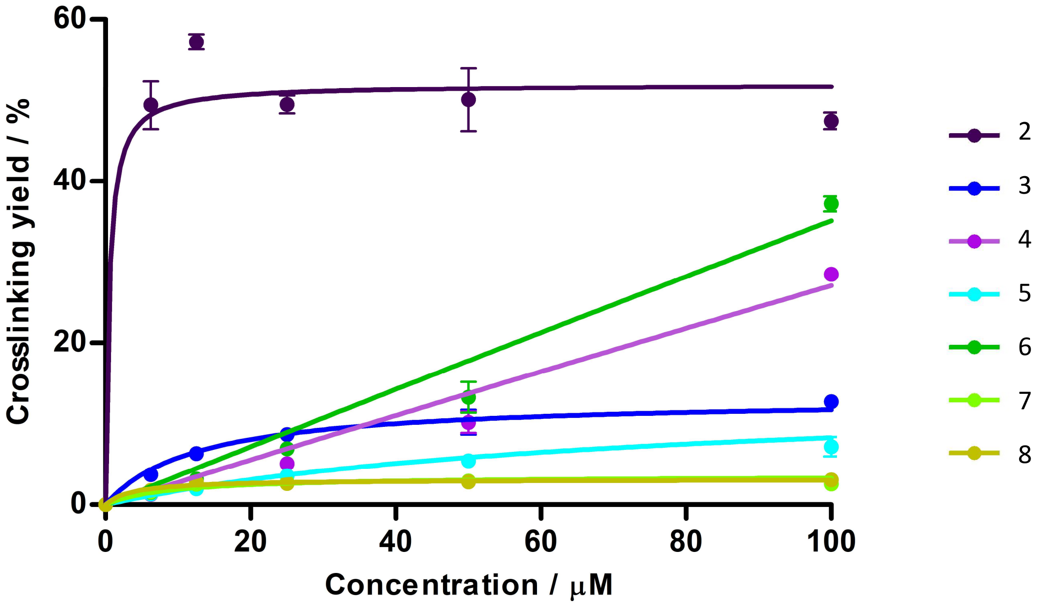

Figure S6. Concentration-response for the 7 hit HTC-PhABits. The unpurified HTC-PhABits were incubated (PBS, 15 min, 4 °C) with carbonic anhydrase I (1 μM) at varying concentrations and irradiated (10 min, 302 nm) and the crosslinking yield was recorded (data recorded in triplicate). HTC-PhABits concentrations were treated assuming full conversion.

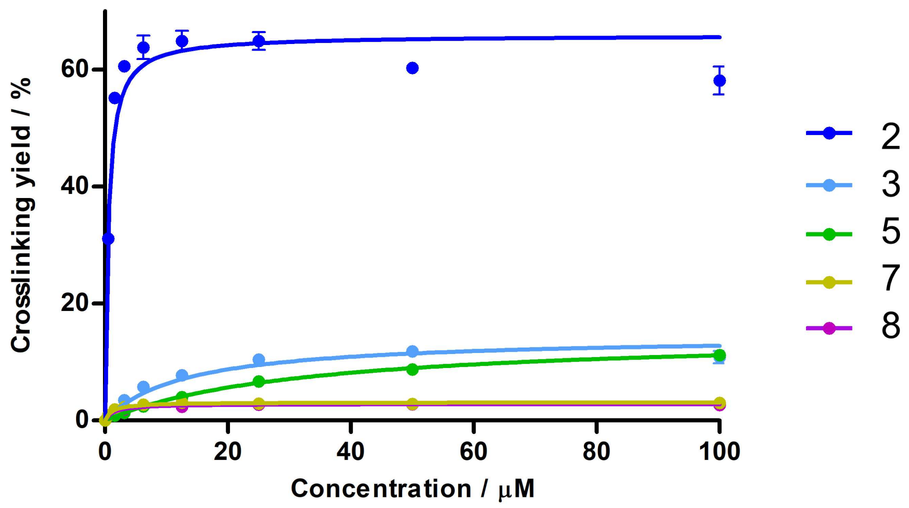

Figure S7. Concentration-response for the 5 hit PhABits (purified) with CAI (0.5 μM). The PhABits were incubated (PBS, 15 min, 4 °C) with carbonic anhydrase I at varying concentrations and *irradiated (10 min, 302 nm) and the crosslinking yield was recorded (data recorded in triplicate).*

Table S4. Crosslinking yields and well positions of the 7 HTC-PhABits (100 µM) with carbonic anhydrase II (1 µM).

HTC-PhABit	Crosslinking yield / %
2	7.8
3	35.1
4	3.7
5	14.8
6	7.4
7	16.6
8	21.9

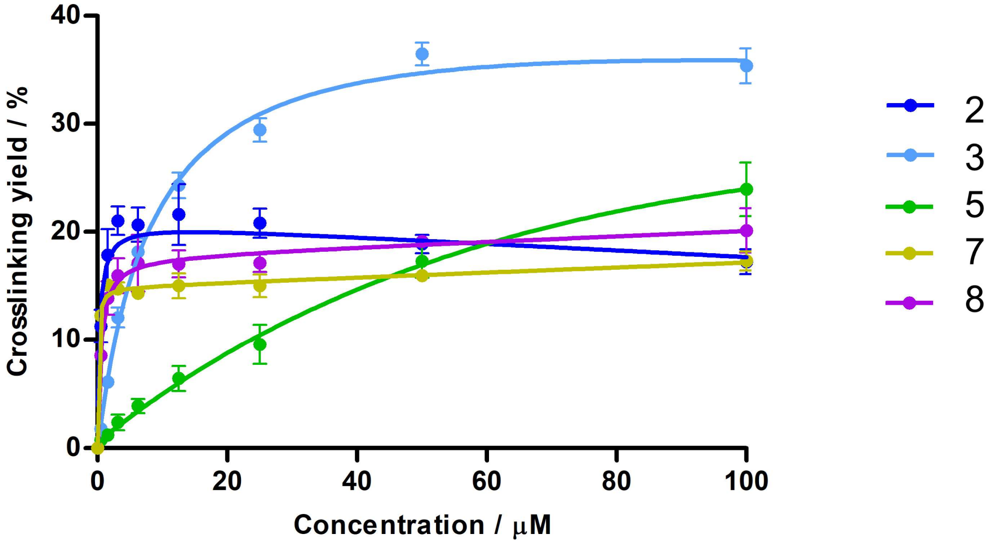

Figure S8. Concentration-response for the 5 hit PhABits (purified) with CAII (0.5 µM). The PhABits were incubated (PBS, 15 min, 4 °C) with carbonic anhydrase II at varying concentrations and *irradiated (10 min, 302 nm) as before and the crosslinking yield was recorded (data recorded in triplicate).*

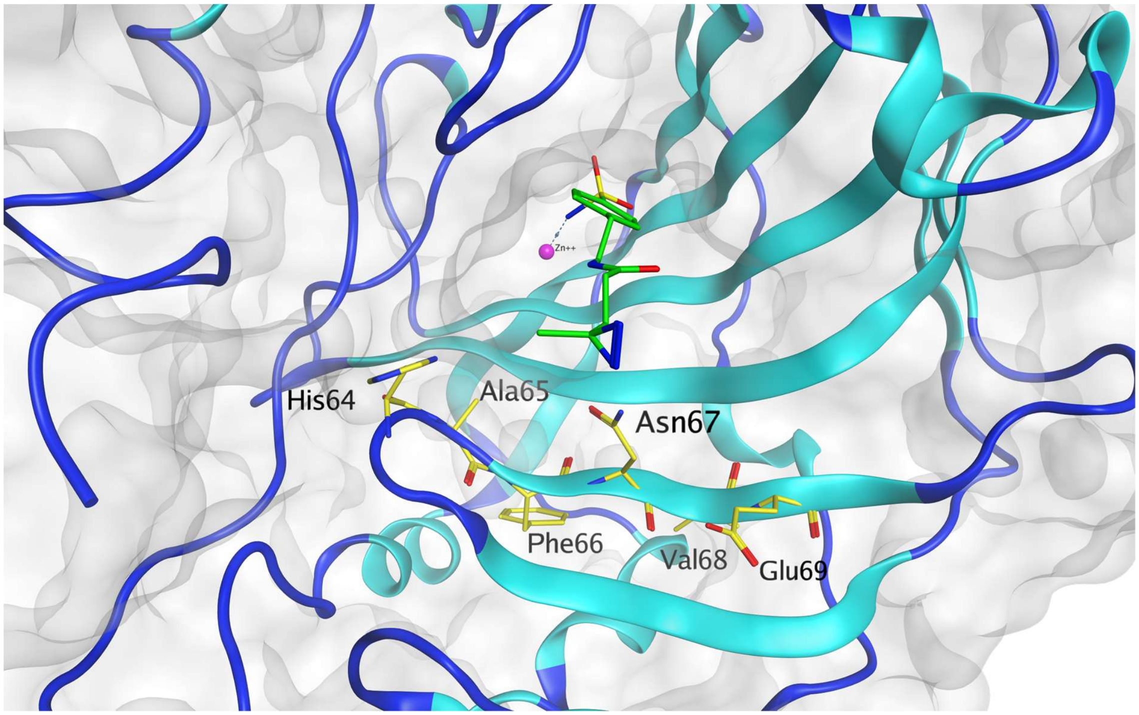

*Figure S9. Docked PhABit **8** in the CAII Zn^{2+} binding pocket (PDB: 3CAJ) highlighting the* $_{65}$AFNV$_{68}$ peptide that remained unmodified by the PhABit*.*

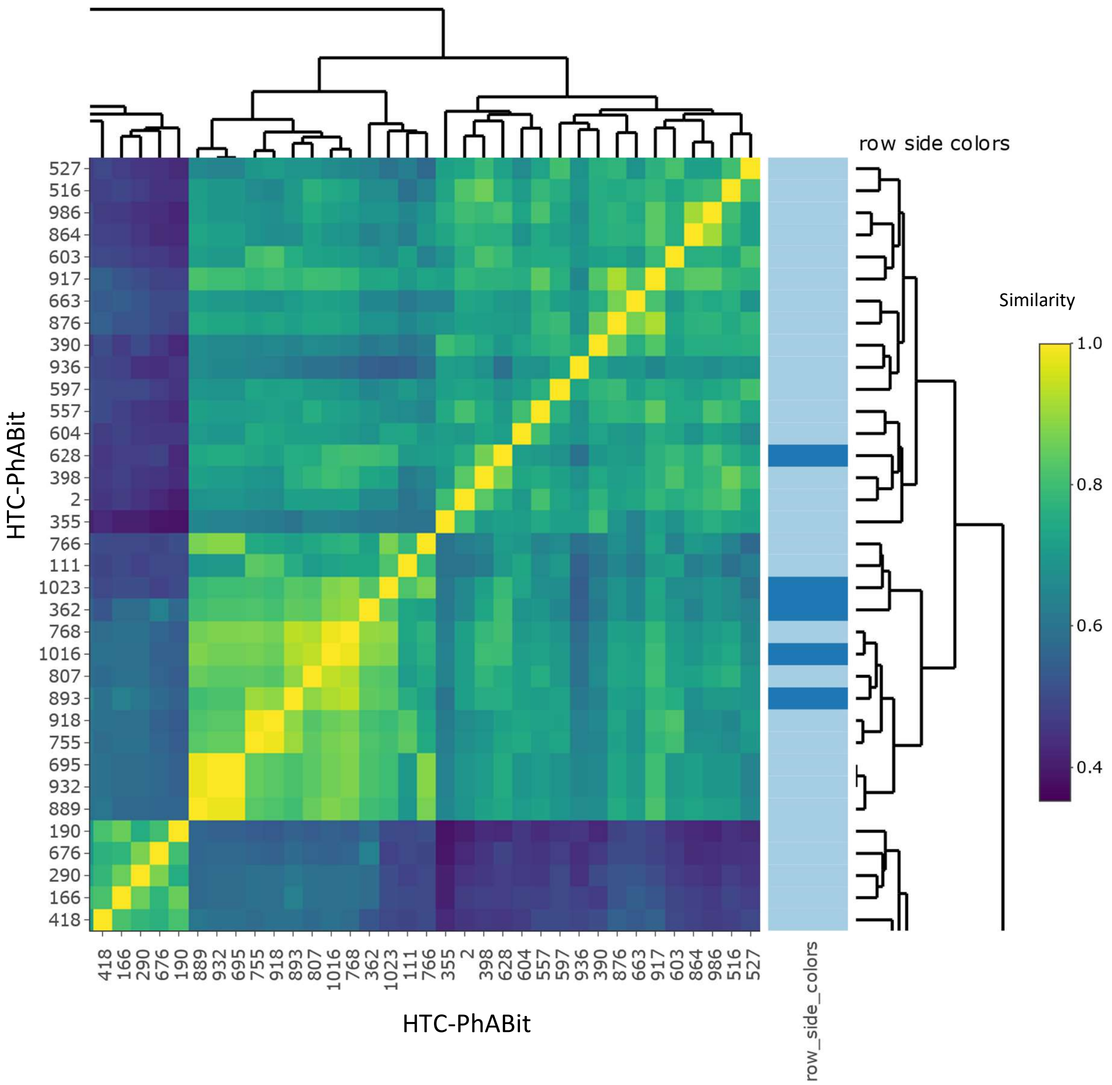

Figure S10. Zoomed in portion of the heatmap of similarity across the 1073-membered HTC-PhABit library. The side column indicates crosslinking to carbonic anhydrase I (dark blue = >1.5% crosslinking). Numbering unique to the heatmap, please see Table S3 overleaf.

Table S5. Smiles of the HTC-PhABits within the heat spot containing the sulfonamide hits. Numbering unique to the heatmap.

HTC-PhABit	SMILES	Crosslinking yield / %
**2**	CC1(CCC(=O)N2CC3CN(CC3C2)S(=O)(=O)C2CC2)N=N1	0.1
**111**	CC(NC(=O)CCC1(C)N=N1)c1ccccc1NS(C)(=O)=O	0.0
**355**	CC1(CCC(=O)N2CC[C@H]3[C@H]2C2(CCC2)C(=O)N3S(C)(=O)=O)N=N1	0.0
**362**	Cc1cc(cc(Cl)c1CNC(=O)CCC1(C)N=N1)S(N)(=O)=O	51.1
**390**	CC1(CCC(=O)N2CC[C@@]3(CCCN3S(C)(=O)=O)[C@@H](O)C2)N=N1	0.0
**398**	CC1(CCC(=O)N2CCc3sc(cc3C2)S(N)(=O)=O)N=N1	1.0
**516**	CC1(CCC(=O)N2CCN(CC2)S(=O)(=O)c2c[nH]cn2)N=N1	0.0
**527**	CC1(CCC(=O)N2CCc3onc(CNS(C)(=O)=O)c3C2)N=N1	0.0
**557**	CCCCS(=O)(=O)N1C[C@H](NC(=O)CCC2(C)N=N2)[C@H](C1)C1CC1	0.0
**597**	CCc1ccc(CN(CCS(=O)(=O)NC)C(=O)CCC2(C)N=N2)o1	0.0
**603**	CC1(CCC(=O)N2CCCc3ccc(NS(C)(=O)=O)cc3C2)N=N1	0.7
**604**	CC(NC(=O)CCC1(C)N=N1)c1cccc(c1)N1CCCS1(=O)=O	0.0
**628**	CC1Cc2ccc(cc2CN1C(=O)CCC1(C)N=N1)S(N)(=O)=O	2.0
**663**	CC1(CCC(=O)NC2CCN(CC2)S(=O)(=O)N2CCOCC2)N=N1	0.1
**695**	CC1(CCC(=O)NCc2cccc(c2)S(C)(=O)=O)N=N1	0.0
**755**	CC1(CCC(=O)NCc2ccc(NS(C)(=O)=O)cc2)N=N1	0.0
**766**	CC(NC(=O)CCC1(C)N=N1)c1ccc(cc1)S(C)(=O)=O	0.0
**768**	CC1(CCC(=O)NCc2cccc(c2)S(N)(=O)=O)N=N1	0.0
**807**	CC1(CCC(=O)NCCNS(=O)(=O)c2ccccc2)N=N1	0.5
**864**	CN(C)S(=O)(=O)N1CCN(CC1)C(=O)CCC1(C)N=N1	0.0
**876**	CC1(CCC(=O)NCC2CN(CCO2)S(C)(=O)=O)N=N1	0.0
**889**	CC1(CCC(=O)NCCc2ccc(cc2)S(C)(=O)=O)N=N1	0.0
**893**	CC1(CCC(=O)NCc2cccc(CS(N)(=O)=O)c2)N=N1	4.9
**917**	CC1(CCC(=O)NCC2CCCN(C2)S(C)(=O)=O)N=N1	0.4
**918**	CC1(CCC(=O)NCc2cccc(NS(C)(=O)=O)c2)N=N1	0.2
**932**	CC1(CCC(=O)NCc2ccc(cc2)S(C)(=O)=O)N=N1	0.5
**936**	COCCN(C1CCS(=O)(=O)C1)C(=O)CCC1(C)N=N1	0.7
**986**	CCS(=O)(=O)N1CCN(C(C)C1)C(=O)CCC1(C)N=N1	0.3
**1016**	CC1(CCC(=O)NCCc2ccc(cc2)S(N)(=O)=O)N=N1	2.5
**1023**	CC(NC(=O)CCC1(C)N=N1)c1cccc(c1)S(N)(=O)=O	10.3

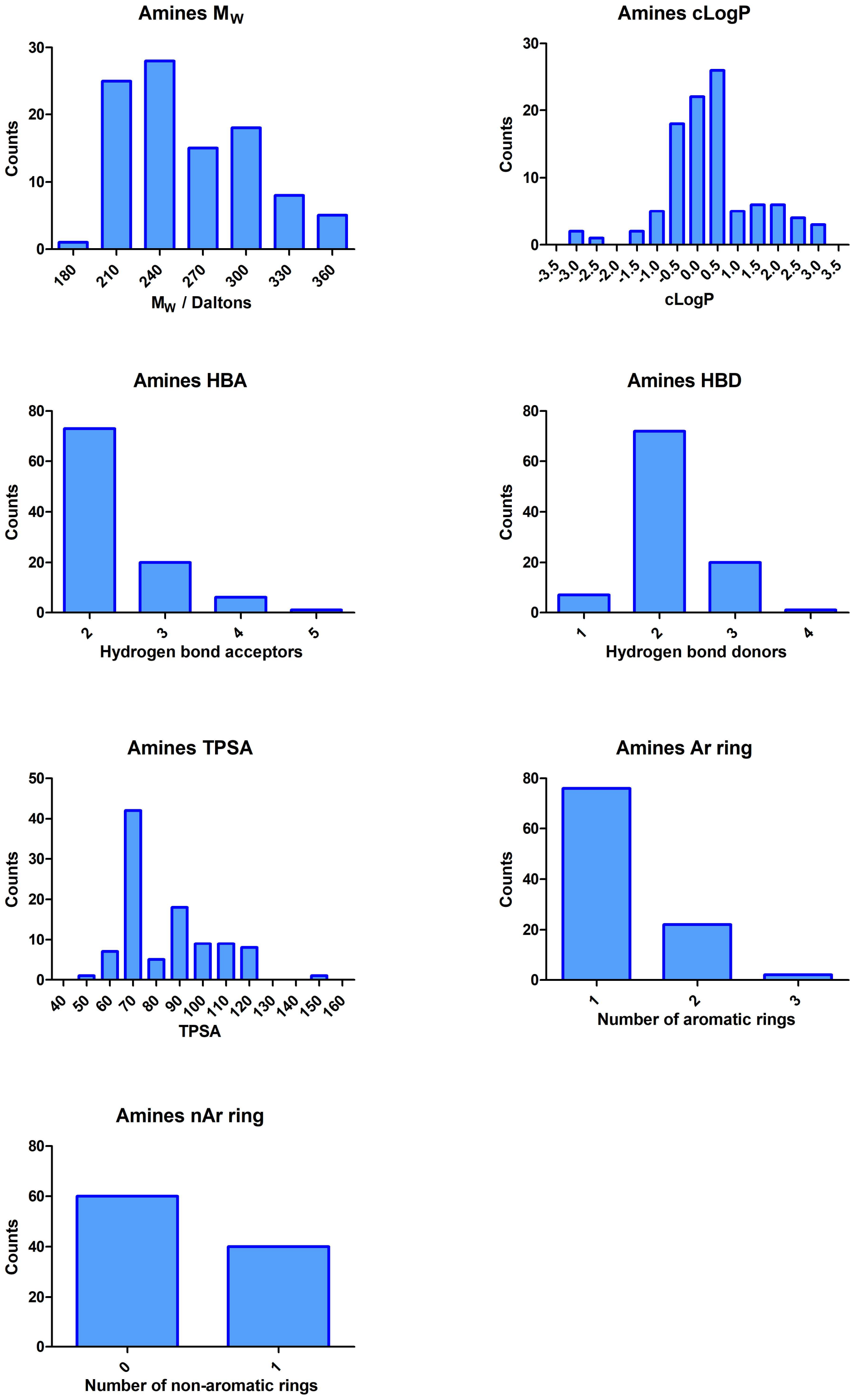

Figure S11. Binned properties of the 100 membered sulfonamide focussed amine library. Free 'uncapped' amines were subjected to the analysis.

2. General Methods

2.1 Solvents, reagents and consumables

Solvents were anhydrous and reagents purchased from commercial suppliers were used as received.

Protein stock solutions used:

- Carbonic anhydrase I from human erythrocytes (Sigma Aldrich, CAS: 9001-03-0, C4396-25MG, Lot: SLBT8230) supplied as a white powder (M_W 28781 Da)
- Carbonic anhydrase II human, recombinant, expressed in *E. coli* (Sigma Aldrich, C6624-500UG, Lot: 069M4082V) supplied as a solution (34.19 µM, M_W 29246 Da) in 20 mM Tris, pH 7.5, with 150 mM NaCl.
- 6H-Tev-BRD4-BD1 (44-168) was produced as part of the GSK/GenScript collaboration and supplied as a solution (676 µM, M_W 15083 Da) in 10 mM HEPES, pH 7.5, with 100 mM NaCl

Plates used:

- Greiner 384 white low volume plates (#784075)
- Greiner 384 PP F-bottom plates (#781201)
- Labcyte ECHO Qualified 384LDV Plus (LPL-0200)

2.2 Irradiation

Irradiation was carried out using an Analytik Jena CL-1000 Ultraviolet Crosslinker (8 Watt, P/N 95-0230-02 with UV-B bulbs, P/N 34-0042-01) at 302 nm in Greiner 384 white low volume plates (#784075). Following irradiation plates were sealed with C.A.S plates seals (#12812447) prior to analysis.

2.3 Nuclear magnetic resonance (NMR) spectroscopy

Proton (^{1}H) and carbon (^{13}C) spectra were recorded in deuterated solvents at 30 °C (variable temperature (VT) spectra record high temperature spectra at 100 °C) using the standardised pulse methods on the Bruker AV-400/600 (^{1}H = 400 MHz, ^{13}C = 101/151 MHz) spectrometer and referenced to residual undeuterated solvent. Chemical shifts are reported in ppm and referenced to tetramethylsilane (TMS) or the following solvent peaks: $CDCl_3$ (^{1}H = 7.27 ppm, ^{13}C = 77.0 ppm), or d_6-

DMSO (^{1}H = 2.50 ppm, ^{13}C = 39.5 ppm). Spectra were processed using ACD/Spectrus Processor 2017.2. Peak assignments chosen based on chemical shifts, integrations and coupling constants, considering 2D analyses where necessary. Coupling constants are quoted to the nearest 0.01 Hz and multiplicities described as either singlet (s), doublet (d), triplet (t), quartet (q), quintet (quin), sextet (sxt), septet (sept), broad (br.) and multiplet (m).

2.4.1 Liquid chromatography-mass spectrometry (LC-MS) for small molecules

Liquid chromatography-mass spectrometry was carried out on a Waters® Acquity UPLC instrument equipped with a CSH Acquity UPLC C18 column (internal diameter: 50 mm × 2.1 mm, packing diameter: 1.7 µm) at 40 °C with a 0.3 µL injection volume. The UV detection was a summed signal from wavelengths between 210 nm and 350 nm. Mass detection was performed with Alternate-scan Positive and Negative Electrospray on a Waters QDA instrument, with a scan range of 100–1000 Da and a scan frequency of 5 Hz.

Low pH: Sample was eluted using a gradient shown in Table S6 with a flow rate of 1.0 mL/min. Solvent A (0.1% *v/v* solution of formic acid in water) and solvent B (0.1% *v/v* solution of formic acid in acetonitrile).

Table S6. Low pH gradient for LC-MS analysis

**Time / min**	**Flow Rate / mL/min**	**Solvent A /%**	**Solvent B /%**
0.0	1	97	3
1.5	1	3	97
1.9	1	3	97
2.0	1	98	2

High pH: Sample was eluted using a gradient shown in Table S7 with a flow rate of 1.0 mL/min. Solvent A (10 mM ammonium bicarbonate in water adjusted to pH 10 with ammonia solution) and solvent B (acetonitrile).

Table S7. High pH gradient for LC-MS analysis

**Time / min**	**Flow Rate / mL/min**	**Solvent A / %**	**Solvent B / %**
0.0	1	100	0
0.05	1	100	0
1.5	1	3	97
1.9	1	3	97
2.00	1	100	0

2.4.2 LC-MS of HTC-PhABit library

For LC-MS of the HTC-PhABit library directly from the Greiner 384 PP F-bottom plate (#781201) the following protocol was used: Liquid chromatography-mass spectrometry was carried out on a Waters® Acquity UPLC instrument equipped with a BEH Acquity UPLC C18 column (internal diameter: 50 mm × 2.1 mm, packing diameter: 1.7 µm) at 40 °C with a 0.2 µL injection volume. The UV detection was a summed signal from wavelengths between 210 nm and 350 nm. Mass detection was performed with Alternate-scan Positive and Negative Electrospray on a Waters SQD2 instrument, with a scan range of 100–1000 Da and a scan frequency of 5 Hz. A HpH method was used and the sample was eluted using a gradient shown in Table S7 with a flow rate of 1.0 mL/min. Solvent A (10 mM ammonium bicarbonate in water adjusted to pH 10 with ammonia solution) and solvent B (acetonitrile).

2.5 Mass directed automated preparative HPLC (MDAP)

Mass directed Autoprep HPLC was carried out on a Waters® Xselect instrument equipped with a CSH C18 column (internal diameter: 150 mm × 30 mm, packing diameter: 5 µm) at ambient temperature with a 1 mL injection volume. The DAD detection was a summed signal from wavelengths between 210 nm and 350 nm. Mass detection was performed with alternate-scan Positive and Negative Electrospray on a Waters QDA instrument, with a scan range of 100–1000 Da and a scan frequency of 4.3 Hz. Two procedures were used:

Low pH: C18 column (150 mm × 30 mm, 5 µm packing diameter, 40.0 mL/min flow rate) using a gradient elution at ambient temperature with the mobile phases of 0.1% *v/v* solution of formic acid in water (solvent A) and 0.1% *v/v* solution of formic acid in acetonitrile (solvent B).

High pH: C18 column (150 mm × 30 mm, 5 µm packing diameter, 40.0 mL/min flow rate) using a gradient elution at ambient temperature using mobile phases of water with 10 mM ammonium bicarbonate adjusted to pH 10 with ammonia solution (solvent A) and acetonitrile (solvent B).

The gradient of acetonitrile required to elute product was determined by the LC-MS retention time. The methods were selected dependent on the retention time of desired material and are shown below (Table S8) and an exemplar gradient for Method B (Table S9).

Table S8. MDAP methods

Method	Flow rate / mL/min	% Acetonitrile	LC-MS t_R / min
A	40	0–30	0.40–0.65
B	40	15–55	0.65–0.90
C	40	30–85	0.90–1.16
D	40	50–99	1.16–1.40
E	40	80–99	1.40–2.00

Table S9. MDAP method B solvent gradient

Time / min	Flow rate / mL/min	Solvent A / %	Solvent B / %
0.0	40	85	15
3.0	40	85	15
12.0	40	45	55
12.5	40	45	55
13.0	40	1	99
17.0	40	1	99

2.6 Infrared Spectroscopy

IR spectra were recorded using a PerkinElmer® Spectrum Two FT-IR machine. Absorption maxima (ν_{max}) are reported in wavenumbers (cm^{-1}) for peaks outside of the fingerprint region.

2.7 Melting Point

Melting points were recorded on a BUCHI Melting Point M-565 apparatus.

2.8 High-resolution mass spectrometry

High-resolution mass spectra were recorded on a Waters® Acquity UPLC instrument equipped with a BEH (HpH method)/CSH (low pH method) C18 column (internal diameter: 100 mm × 2.1 mm, packing diameter: 1.7 µm) at 50 °C with a 0.2 µL injection volume. The UV detection was a summed signal from wavelengths between 210 nm and 500 nm. Mass detection was performed with Alternate-scan Positive and Negative Electrospray on a Waters XEVO G2-XS QToF instrument, with a scan range of 100–1200 Da. Two procedures were used:

Low pH: Gradient elution using mobile phases of water with 0.1% *v/v* solution of formic acid in water (solvent A) and 0.1% *v/v* solution of formic acid in acetonitrile (solvent B) (Table S10).

High pH: Gradient elution using mobile phases of water with 10 mM ammonium bicarbonate adjusted to pH 10 with ammonia solution (solvent A) and acetonitrile (solvent B) (Table S11).

Table S10. HRMS low method solvent gradient

Time / min	Flow rate / mL/min	Solvent A / %	Solvent B / %
0.0	0.8	95	5
8.5	0.8	7	93
9.0	0.8	7	93
9.5	0.8	95	5
10.0	0.8	95	5

Table S11. HRMS HpH method solvent gradient

Time / min	Flow rate / mL/min	Solvent A / %	Solvent B / %
0.0	0.8	99	1
0.5	0.8	99	1
17.0	0.8	10	90
18.5	0.8	10	90
19.0	0.8	99	1
20.0	0.8	99	1

2.9 TLC and Flash column chromatography

TLC was carried out using polyester-backed precoated silica plates (0.2 mm particle size). Spots were visualised under UV light of λ_{max} = 254 nm or 365 nm or by heating with potassium permanganate stain.

Column chromatography was carried out using the Teledyne ISCO Combi*Flash*® R*f*+ apparatus with Redi*Sep*® silica cartridges. Compounds were visualised by UV (λ = 254 and 280 nm)

2.10 Intact Protein LC-MS

Intact protein masses were recorded by LC-MS using:

- An Agilent G6224 time-of-flight (ToF) Accurate Mass Series mass spectrometer, interfaced with an Agilent 1200 series liquid chromatography and sample handling system. The protein

sample was injected using an Agilent 1200 series AutoSampler (Model No. G1367B) with a 10 µL injection volume and maintained at a temperature of 10 °C. Chromatography was carried out on an Agilent Bio-HPLC PLRP-S (1000 Å, 5 µm × 50 mm × 1.0 mm, PL1312-1502) reverse phase HPLC column at 70 °C. Using an Agilent 1200 series binary pump system (Model No. G1312B) the sample was eluted at 0.5 mL/min using a gradient system from Solvent A (water, 0.2% (*v/v*) formic acid) to Solvent B (acetonitrile, 0.2% (*v/v*) formic acid) according to the conditions described in Table S12. The eluent was injected directly into an Agilent ToF mass spectrometer (Model No. G6224A) using a dual ESI source and scanning between 600-3200 Da with a scan rate of 1.03 s in positive mode. The following MS parameters were used: capillary voltage limit – 4200 V; desolvation temperature – 340 °C; drying gas flow – 8.0 L/min.

Table S12. Intact protein LC-MS solvent gradient

Time (min)	Flow rate / mL/min	Solvent A / %	Solvent B /%
**0**	0.5	80	20
**0.6**	0.5	80	20
**0.61**	0.5	50	50
**1.8**	0.5	0	100
**2.0**	0.5	0	100
**2.01**	0.5	80	20

- An Agilent G6230B time-of-flight (ToF) Accurate Mass Series mass spectrometer, interfaced with an Agilent 1290 infinity II series column oven (G7116B) and an Agilent 1290 infinity II series liquid chromatography high speed binary pump (G7120A). The protein sample was injected using an Agilent 1290 infinity II series multisampler with dual needles (Model No. G7167B) with a 10 µL injection volume and maintained at a temperature of 4 °C. Chromatography was carried out on an Agilent Bio-HPLC PLRP-S (1000 Å, 5 µm × 50 mm × 1.0 mm, PL1312-1502) reverse phase HPLC column at 70 °C. The sample was eluted at 0.5 mL/min using a gradient system from Solvent A (water, 0.2% (*v/v*) formic acid) to Solvent B (acetonitrile, 0.2% (*v/v*) formic acid) according to the conditions described in Table S13. The eluent was injected directly into an Agilent ToF mass spectrometer (Model No. G6230B) using a dual AJS ESI source and scanning between 600-3200 Da with a scan rate of 1.20 s in positive mode. The following MS parameters were used: capillary voltage limit – 4000 V; desolvation temperature – 350 °C; drying gas flow – 10 L/min.

Table S13. Intact protein LC-MS solvent gradient

Time (min)	Flow rate / mL/min	Solvent A / %	Solvent B /%
**0.6**	0.5	80	20
**0.61**	0.5	50	50
**1.0**	0.5	0	100
**1.2**	0.5	0	100
**1.21**	0.5	80	20

Data acquisition was carried out in 2 GHz Extended Dynamic range mode. Spectra were processed using Mass Hunter Qualitative Analysis™ B06.00 (Agilent) software with the Maximum Entropy method employed. The total ion chromatograms (TIC) were extracted (region containing protein) and the summed scans were deconvoluted (using a maximum entropy algorithm) over a *m/z* range with an expected mass range dependent on the protein.

Table S14. Deconvolution conditions for target proteins examined in this work

Protein	*m/z* range	Expected mass range
**Carbonic Anhydrase I**	800-2000	28000-32000
**Carbonic Anhydrase II**	800-2000	28000-32000
**BRD4-BD1**	900-2000	14000-17000

The deconvoluted spectra were exported as csv files and analysed using R Studio software (Version 3.6.3) to generate pdf files of the spectra. The median of the protein only controls were subtracted from the sample spectra to remove baseline signal. The peak height for unmodified protein and labelled protein were recorded and used to calculate percentage photocrosslinking using the equation:

$$\%\,crosslinking = \frac{height\ of\ modification\ peak}{height\ of\ modification\ peak + height\ of\ parent\ peak} \times 100$$

2.11 Centrifuge

Plates were centrifuged using a Sorvall Legend RT (401198833) model at 1000 rpm for 1 minute.

2.12 Molecular docking

Virtual molecular docking of PhABit **8** was carried out in Molecular Operating Environment (MOE) (Version 2019.0101). PDB file 3CAJ (human CAII complexed with ethoxzolamide) was used. The docking tool was used to map PhABit **8** in the Zn^{2+} binding site using ethoxzolamide as a template. The methods used were Placement: Triangle Matcher; Score: London dG and Refinement: Rigid Receptor; Score: GBVI/WSA dG.

3. Library synthesis

3.1 Amine selection

Amines were initially selected to include those alkyl amines from the original PhABit library.[1] The rest of the amines in the HTC-PhABit library were then chosen following a molecular clustering algorithm.

A diverse set of aliphatic amines containing only one amine group was to be selected in addition to the aliphatic amines from the original PhABit library.[1] The starting set for the selection was the solution sample store of the GSK, however, compounds were also checked for the availability in eMolecules and Enamine building block databases. The selection was done using BioVia Pipeline Pilot [BIOVIA, Dassault Systèmes, Pipeline Pilot, 20.1.0.2208, San Diego: Dassault Systèmes, 2020]. Initial selection criteria were applied: GSK compounds having at least one sample with concentration 10 mM in DMSO or above and at least 150 µL sample size. Unstable compounds and compounds with other liabilities were removed using proprietary GSK filters. Further selection criteria were then applied: $150<M_W<250$, BioByte cLogP<9 and only 1 aliphatic amine group.[2] The structures were tagged based on 6 aliphatic amine types, differentiating based on primary/secondary, hindered/non-hindered and cyclic/non-cyclic. The amines were also tagged by the calculated pKa using ChemAxon pKa.[3] All amines having acidic pKa<7 and amines with secondary hindered linear aliphatic amines were excluded. A ChemAxon LibMCS clustering was performed on the remaining *ca*. 4000 molecules.[4] Since the aim of the current selection was to choose compounds which can have analogues, all compounds were excluded which did not belong to a cluster with at least 4 members. The remaining *ca*. 3500 molecules were joined with the list of previous amines from the original PhABit library.[1] A diverse selection was then performed using Diverse Molecules component from Pipeline Pilot considering the already selected molecules and using ECFP4 fingerprints resulting 1073 molecules.[5]

The selected amines (10 mM) were ordered from GSK's solution stores in Greiner 384 PP F-bottom plates (#781201). Following small molecule LC-MS directly from the plates to assess the amine's initial purity the HTC reactions were undertaken:

3.2.1 HTC-PhABits group A (final concentration 5 mM)

Two Greiner PP 384-well plates were charged with 678 amines (10 mM, 37 µL). LC-MS analysis of the amine starting materials was conducted prior to reaction (**SI section 2.4.2**). A stock solution of compound **1** and NEM was prepared in DMSO such that their respective concentrations were 11 and 30 mM. To the plated amines, the stock solution (37 µL) was added, meaning the final concentration of the HTC-PhABits were 5 mM in 74 µL. The plates were sealed, centrifuged (1 min, 1000 rpm) and left standing at room temperature for 24 h. After the reaction the plates were subjected to small molecule LC-MS analysis to identify and quantify product formation.

To quench the reaction mixture, a 5.5 µL aliquot of the reaction mixture was removed and added to new Labcyte 384LDV plates (LPL-0200). A hydroxylamine solution (50% wt in H_2O, 30.3 M) was diluted 1 in 10 to afford a 3.03 M hydroxylamine solution which was dispensed (5 µL) across an empty Labcyte 384LDV plate (LPL-0200). Using a Labcyte ECHO© 555 Liquid Handler, 20 nL of the hydroxylamine solution was added to each reaction mixture well such that its final concentration was 11 mM. The plates were sealed and left to stand at room temperature for 1 h. The plates were stored in a -20 °C freezer.

3.2.2 HTC-PhABits group B (final concentration 8 mM)

Two Greiner PP 384-well plates were charged with 395 amines (10 mM, 17 µL). LC-MS analysis of the amine starting materials was conducted prior to reaction (**SI section 2.4.2**). A stock solution of compound **1** and NEM was prepared in DMSO such that their respective concentrations were 44 and 120 mM. To the plated amines, the stock solution (4.3 µL) was added, meaning the final concentration of the HTC-PhABits were 8 mM in 21.3 µL. The plates were sealed, centrifuged (1 min, 1000 rpm) and left standing at room temperature for 24 h. After the reaction the plates were subjected to small molecule LC-MS analysis to identify and quantify product formation.

To quench the reaction mixture, a 5.3 µL aliquot of the reaction mixture was removed and added to new Labcyte 384LDV plates (LPL-0200). A hydroxylamine solution (50% wt in H_2O, 30.3 M) was diluted 1 in 10 to afford a 3.03 M hydroxylamine solution which was dispensed (5 µL) across an empty Labcyte LDV plate (LPL-0200). Using a Labcyte ECHO© 555 Liquid Handler, 30 nL of the hydroxylamine solution

was added to each reaction mixture well such that its final concentration was 17.6 mM. The plates were sealed and left to stand at room temperature for 1 h. The plates were stored in a -20 °C freezer.

4. Screening the HTC-PhABit library

4.1 HTC-PhABit plate preparation

Using a Labcyte ECHO© 555 Liquid Handler 300 nL of the Group A HTC-PhABits (5 mM) were transferred to a Greiner 384 white low volume plates (#784075). Similarly, 190 nL of the Group B HTC-PhABits (8 mM) and 110 nL of DMSO were transferred to a Greiner 384 white low volume plates (#784075). Such that the HTC-PhABit concentrations were all 5 mM (300 nL) prior to protein addition.

4.2 Protein addition and irradiation protocol

For human carbonic anhydrase I (CAI) (M_W 28781 Da), the solid was weighed out (typically ~1-1.5 mg) and dissolved in the appropriate volume of Milli-Q phosphate-buffered saline (PBS) (pH ~7.2) such that its concentration was 100 µM. This stock solution was further diluted in PBS to afford a 1 µM CAI stock solution.

For human carbonic anhydrase II (34.19 µM, M_W 29246 Da), the stock solution was diluted in the appropriate volume of Milli-Q phosphate-buffered saline (PBS) (pH ~7.2) such that its concentration was 1 µM.

To the compound containing (300 nL, 5 mM) Greiner 384 white low volume plates a 15 µL aliquot of protein stock solution (1 µM) was added to the HTC-PhABit containing wells at 0 ^{o}C, meaning the final DMSO composition was 2.0 % (*v/v*) and the final concentration of HTC-PhABit was 100 µM. To the control and blank wells respectively, protein stock solution (15 µL, 1 µM) and MQ water (15 µL) were added. The plate was equilibrated at 0 ^{o}C for 15 minutes before irradiation with UV light (302 nm, **SI section 2.2**) for 10 minutes. The plates were centrifuged (1 min, 1000 rpm). The individual wells were analysed by LC-MS-TOF mass spectrometry (**SI section 2.10**).

4.3 Single shot screen of 1073 HTC-PhABits with CAI

The library of 1073 HTC-PhABits were plated according to **SI section 4.1** and a 1 µM stock solution of CAI was prepared and added across the plates (**SI section 4.2**). The plate was then subjected to UV irradiation (**SI section 4.2**). Protein concentration: 1 µM, HTC-PhABit concentration: 100 µM, DMSO

content 2.0% (*v/v*). The individual wells of the 4 × 384-well plates were analysed by LC-MS-TOF mass spectrometry (**SI section 2.10**). Hits were classed as those HTC-PhABits displaying a baseline subtracted crosslinking yield >1.5%.

5. Follow-up studies: competition, concentration-response, LC-MS/MS

5.1 Competition studies with ethoxzolamide

The 7 HTC-PhABit hits were plated into a Greiner 384 white low volume plate such that their concentration was 5 mM (300 nL) according **SI section 4.1**. A 100 µM CAI stock was prepared (**SI section 4.2**). The 100 µM CAI stock solution was diluted into 2 x 1 mL solutions in PBS, such that the final concentration was 1 µM. To one, a 100 mM stock of ethoxzolamide (purchased from Sigma Aldrich) in DMSO was added (1 µL) such that its final concentration was 100 µM. To the second stock, DMSO was added (1 µL). To the compounds in a Greiner 384 white low volume plate was added either a 15 µL aliquot of protein stock solution (1 µM) + ethoxzolamide or protein stock solution (1 µM) + DMSO at 0 ^{o}C, meaning the final DMSO composition was 2.1 % (*v/v*) and the final concentration of HTC-PhABit was 100 µM. To the control and blank wells respectively, protein stock solution (15 µL, 1 µM) and MQ water (15 µL) were added. The plate was equilibrated at 0 ^{o}C for 15 minutes before irradiation with UV light (302 nm) for 10 minutes. The plates were centrifuged (1 min, 1000 rpm). The individual wells were analysed by LC-MS-TOF mass spectrometry (**SI section 2.10**).

5.2 Concentration response procedures

5.2.1 Concentration-response with HTC-PhABits

To determine the dissociation constant (K_D) of 7 HTC-PhABit hits, identified from the screen against CAI, the unpurified hits were irradiated with CAI at varying concentrations. Using a Labcyte ECHO© 555 Liquid Handler the HTC-PhABits were diluted with different ratios of DMSO (in triplicate) in Greiner 384 white low volume plates according to the table below such that the total volume was 300 nL (Table S15).

Table S15. Dilutions of the HTC-PhABits with DMSO for concentration-response studies

		**Group A HTC-PhABits (5 mM)**		**Group B HTC-PhABits (8 mM)**	
HTC-PhABit concentration in 15.3 µL / mM	HTC-PhABit concentration in 300 nL / mM	PhABit volume /nL	DMSO volume / nL	PhABit volume /nL	DMSO volume / nL
100	5	300	0	190	110
50	2.5	150	150	95	205
25	1.25	75	225	47.5	252.5
12.5	0.625	37.5	262.5	25	275
6.25	0.313	20	280	12.5	287.5

A 1 µM stock solution of CAI was prepared according to **SI section 4.2.** A 15 µL aliquot of the CAI (1 µM) stock solution was added and irradiation was carried out according to **SI section 4.2**. Protein concentration: 1 µM, HTC-PhABit concentration: 100-6.25 µM, DMSO content 2 % (*v/v*), n=3. The individual wells of the plates were analysed by LC-MS-TOF mass spectrometry (**SI section 2.10**).

The crosslinking *vs* concentration for each HTC-PhABit was plotted on GraphPad Prism 5.0.4, each data point was recorded in triplicate and the mean and standard deviation was calculated and plotted. In some instances, for comparison, crosslinking yields were normalised to allow comparison.

5.2.2 Concentration-response with purified PhABits

The five purified sulfonamide hits (**2**, **3**, **5**, **7**, **8**, **SI section 7.3**) were irradiated with both CAI and CAII at varying concentrations to determine their respective dissociation constants (K_D). The PhABits were dissolved in DMSO such that their concentrations were 10 and 1 mM. The 10 and 1 mM PhABit stock solutions were aliquoted (5 µL) into a Labcyte ECHO Qualified 384LDV Plus plate (LPL-0200). Using a Labcyte ECHO© 555 Liquid Handler the PhABits were diluted with different ratios of DMSO (in triplicate) in a Greiner 384 white low volume plate according to the table below such that the total volume was 150 nL (Table S16). Stock solutions of CAI and CAII (0.5 µM) were prepared according to **SI section 4.2**. A 15 µL aliquot of the CAI/CAII (0.5 µM) stock solution was added and irradiation was carried out according to **SI section 4.2**. Protein concentration: 0.5 µM, HTC-PhABit concentration: 100-0.5 µM, DMSO content 1 % (*v/v*), n=3. The individual wells of the plates were analysed by LC-MS-TOF mass spectrometry (**SI section 2.10**).

The crosslinking *vs* concentration for each HTC-PhABit was plotted on GraphPad Prism 5.0.4, each data point was recorded in triplicate and the mean and standard deviation was calculated and plotted. In some instances, for comparison, crosslinking yields were normalised to allow comparison.

Table S16. Dilutions of the purified PhABits with DMSO for concentration-response studies

PhABit concentration in 15.15 µL / µM	PhABit concentration in 150 nL / mM	PhABit volume / nL	DMSO volume / nL	PhABit stock to ECHO dispense from / mM
100	10	150	0	10
50	5	75	75	10
25	2.5	37.5	112.5	10
12.5	1.25	18.75	131.25	10
6.25	0.625	93.75	56.25	1
3.13	0.313	46.95	103.05	1
1.56	0.156	23.4	126.6	1
0.5	0.05	7.5	142.5	1
0	0	0	150	NA

5.3 Site of crosslinking LC-MS/MS studies

PhABits **3, 5, 7** and **8** were dissolved in DMSO such that their concentrations were 10 mM. The 10 mM PhABit stock solutions were aliquoted (5 µL) into a Labcyte ECHO Qualified 384LDV Plus plate (LPL-0200). Using a Labcyte ECHO© 555 Liquid Handler the PhABits (150 nL) were dispensed into a Greiner 384 white low volume plate. A stock solution of CAII (2 µM) were prepared according to **SI section 4.2**. A 15 µL aliquot of the CAII (2 µM) stock solution was added and irradiation was carried out according to **SI section 4.2**. Protein concentration: 2 µM, HTC-PhABit concentration: 100 µM, DMSO content 1 % (*v/v*). A second plate was prepared in the same way which was not subjected to 302 nm irradiation.

Irradiated and non-irradiated samples for each compound were processed for LC-MS/MS analysis. Samples (1 µg) were separated by bis-tris sodium dodecyl sulfate–polyacrylamide gel electrophoresis (SDS-PAGE) to remove the excess of unbound compound alongside a SeeBlue protein ladder. Gel was stained with colloidal Comassie InstantBlue and bands corresponding to CAII at ~30 kDa were excised and transferred to LoBind microtubes. Gel pieces were destained by addition of 400 µL of ammonium bicarbonate pH 8.0/acetonitrile (1:1 *v:v*) and agitated for 30 min at room temperature. The destain solution was discarded and the samples were reduced by addition of 100 µL of 10 mM TCEP (in 100 mM ammonium bicarbonate pH 8.0) for 30 min at 65 °C. The reducing solution was discarded and samples were alkylated by addition of 100 µL of 10 mM iodoacetamide (in 100 mM ammonium bicarbonate pH 8.0) for 30 min at room temperature in the dark. Samples were washed sequentially with 400 µL of 25 mM ammonium bicarbonate pH 8.0, 400 µL of 25 mM ammonium bicarbonate pH 8.0/acetonitrile (1:1, *v:v*) and 400 µL of acetonitrile with brief agitation between each wash step. Samples were digested for 16 h at 37 °C with trypsin (Promega) at a 1:10 enzyme to sample ratio in 40 µL of 25 mM ammonium bicarbonate pH 8.0. The resulting supernatant was transferred to new LoBind microtubes and peptides were extracted by washing the gel pieces with 50 µL of acetonitrile and brief vortexing. This was repeated twice and pooled with the supernatant. The extracted peptide solutions were snap frozen on dry ice before being dried by SpeedVac. Dried peptides were resuspended in 0.1% formic acid, 0.05% trifluoroacetic acid, agitated briefly before centrifugation (4000 × g, 5 min) and transferred to autosampler vials.

Digested samples were injected on an Easy-nLC 1000 UHPLC system (Thermo Scientific). The nanoLC was interfaced to a Q-Exactive Hybrid Quadrupole-Orbitrap Mass Spectrometer (Thermo Scientific). Tryptic peptides were loaded on a 2 cm × 75 µm Acclaim PepMap 100 C18 trapping column (Thermo Scientific) and separated on a 25 cm × 75 µm, 2 µm particles, PepMap C18, 2 µm particle column (Thermo Scientific) using a 50 min gradient of 2-38% acetonitrile, 0.2% formic acid and a flow rate of

300 nL/min. LC-MS/MS based peptide sequencing was performed by data dependent analysis (DDA) (Full MS 400-1600 Da at 70,000 resolution, MS AGC target 1e6, MS Maximum IT 200 ms, followed by MS/MS top 10 HCD fragmentation, stepped normalized CE 23, 27 and 30 V, isolation window 1.5 *m/z*, fixed first mass 145 *m/z*, 17,500 resolution, MS/MS AGC target 5e4 and MS/MS Maximum IT 200 ms.

Samples were also analyzed using Parallel Reaction Monitoring (PRM) methods targeting the CAII peptide 59-76 modified by compounds **3**, **5**, and **8** (*m/z* 782.3537, 3+ ion) or compound **7** (*m/z* 791.0262, 3+ ion). MS/MS parameters included stepped normalized CE 23, 27 and 30 V, isolation window 1.2 *m/z*, fixed first mass 100 *m/z*, 17,500 resolution, AGC target 2e5 and Maximum IT 250 ms.

Uninterpreted tandem MS spectra were searched for peptide matches against the human sequence for carbonic anhydrase II using the Mascot software (Version 2.6.0) (Matrix Science) with a 5 ppm mass tolerance for peptide precursors and 20 mDa mass tolerance for fragment ions.

Raw files were searched using trypsin as the enzyme with up to 2 missed cleavages and the variable modifications carboamidomethylation (C) and oxidation (M). Oxidation on methionine, carbamidomethylation on cysteine and PhABit-N_2 modification on any residue were allowed as variable modifications. PRM MS/MS spectra were manually validated and annotated.

6. Iterative HTC-PhABit library synthesis and screening

6.1 Amine selection

Amines were selected to include those that had molecular similarity to the initial hit PhABits containing a primary sulfonamide (**2, 3, 5, 7** and **8**).

The similarity seraching ws performed using a BioVia Pipeline Pilot [BIOVIA, Dassault Systèmes, Pipeline Pilot, 20.1.0.2208, San Diego: Dassault Systèmes, 2020] protocol. Initial selection criteria were applied: GSK compounds having at least one sample with concentration 10 mM in DMSO or above and at least 50 µL sample size. Unstable compounds and compounds with other liabilities were removed using proprietary GSK filters. Further selection criteria were then applied: $150<M_W<350$, BioByte cLogP<9 and only 1 aliphatic amine group.[2] Substructure filters were used to create a pool of molecules containing 1 amine group. Tanimoto similarities between the original query compound(s) and the pooled compounds were calculated separately and the compounds were tagged whether they were generated with BioDig molecule generator from the original query compound. The list was provided to the user for processing.

The selected amines (10 mM) were ordered from GSK's solution stores in Greiner 384 PP F-bottom plates (#781201). Following small molecule LC-MS directly from the plates to assess the amine's initial purity the HTC reactions were undertaken:

6.2 Second generation HTC-PhABit library

A single Greiner PP 384-well plate (#781201) was charged with 100 amines (10 mM, 27 µL). LC-MS analysis of the amine starting materials was conducted prior to reaction (**SI section 2.4.2**). A stock solution of compound **1** and NEM was prepared in DMSO such that their respective concentrations were 44 and 120 mM. To the plated amines, the stock solution (6.8 µL) was added, meaning the final concentration of the HTC-PhABits were 8 mM in 33.8 µL. The plates were sealed, centrifuged (1 min, 1000 rpm) and left standing at room temperature for 24 h. After the reaction the plates were subjected to small molecule LC-MS analysis to identify and quantify product formation.

To quench the reaction mixture, a 5.3 µL aliquot of the reaction mixture was removed and added to a new Labcyte 384LDV plate (LPL-0200). A hydroxylamine solution (50% wt in H_2O, 30.3 M) was diluted 1 in 10 to afford a 3.03 M hydroxylamine solution which was dispensed (5 µL) across an empty Labcyte 384LDV plate (LPL-0200). Using a Labcyte ECHO© 555 Liquid Handler, 30 nL of the hydroxylamine solution was added to each reaction mixture well such that its final concentration was 17.6 mM. The

plates were sealed and left to stand at room temperature for 1 h. The plates were stored in a -20 °C freezer.

6.3 Screening the second-generation HTC-PhABit library with CAI

Using a Labcyte ECHO© 555 Liquid Handler the HTC-PhABits were diluted such that their concentration was 5 mM in 300 nL in a Greiner 384 white low volume plates prior to protein addition (**SI section 4.1**). A CAI (1 µM) stock solution in PBS was prepared according to **SI section 4.2**. To the compound containing (300 nL, 5 mM) Greiner 384 white low volume plates a 15 µL aliquot of CAI stock solution (1 µM) was added, incubated and irradiated according to **SI section 4.2** such that final DMSO composition was 2 % (*v/v*) and the final concentration of HTC-PhABit was 100 µM. The individual wells were analysed by LC-MS-TOF mass spectrometry (**SI section 2.10**). Hits were classed as those HTC-PhABits displaying a baseline subtracted crosslinking yield >1.5% (Table S17).

Table S17. Smiles and crosslinking yields of the 52 hit second generation focussed library of HTC-PhABits with carbonic anhydrase I.

**Position**	**SMILES**	**Crosslinking yield / %**
**A02**	CC(N)Cc1cccc(c1)S(N)(=O)=O	2.7
**A03**	NS(=O)(=O)c1ccc(CCNCc2ccco2)cc1	3.6
**A05**	NCc1ccc(cc1)S(N)(=O)=O	7.2
**A07**	CC(N)(Cc1cccc(c1)S(N)(=O)=O)C(O)=O	5.2
**B03**	NS(=O)(=O)c1c(Cl)ccc2CCNCc12	13.2
**B04**	NS(=O)(=O)c1ccc2CCNCc2c1	1.7
**B05**	NS(=O)(=O)c1ccc(CCNCc2ccc(F)cc2)cc1	6.1
**B07**	NS(=O)(=O)c1cc2CCNCc2cc1Cl	13.1
**C03**	COc1ccccc1CNCCc1ccc(cc1)S(N)(=O)=O	4.4
**C04**	NCCNC(=O)\C=C\c1cccc(c1)S(N)(=O)=O	13.6
**C05**	NC1CCN(CC(=O)NCc2ccc(cc2)S(N)(=O)=O)CC1	12.5
**D04**	NCC(=O)Nc1ccc(cc1)S(N)(=O)=O	38.8
**D05**	NS(=O)(=O)c1cccc2CNCCc12	2
**D07**	NCc1ccc(CS(N)(=O)=O)cc1	7.1
**E02**	NS(=O)(=O)c1ccc(CCNCC2CCCCC2)cc1	3.2
**F02**	NCCc1ccc(s1)S(N)(=O)=O	3.5
**F04**	NS(=O)(=O)c1ccc(CCNC(=O)C2CCCNC2)cc1	29.4
**F05**	NS(=O)(=O)c1ccc(CCNc2ncnc3CCNCCc23)cc1	3.4
**F07**	NS(=O)(=O)c1ccc(CCNCCC2CCCC2)cc1	4.5
**G01**	NCCc1ccc(O)c(c1)S(N)(=O)=O	2.7
**G05**	NCc1c(F)cc(cc1F)S(N)(=O)=O	11.8

**H01**	CC1NCCc2ccc(cc12)S(N)(=O)=O	8.6
**H03**	Cc1ccsc1CNCCc1ccc(cc1)S(N)(=O)=O	2.7
**H04**	NS(=O)(=O)c1cccc2CCNCc12	4.2
**H05**	CNCCc1ccc(cc1)S(N)(=O)=O	7.1
**H07**	CCN(CCc1ccc(cc1)S(N)(=O)=O)C(=O)CN	21.6
**I01**	NCCNC(=O)Cc1ccc(c2ccccc12)S(N)(=O)=O	4.8
**I02**	N[C@@H](CCC(=O)Nc1ccc(cc1)S(N)(=O)=O)C(O)=O	41.8
**I03**	CNC(C)c1ccc(cc1)S(N)(=O)=O	7.1
**I04**	CCNCC(=O)Nc1ccc(cc1)S(N)(=O)=O	27.2
**I05**	NCC(O)c1ccc(Cl)c(c1)S(N)(=O)=O	33
**I07**	Cc1ccc(cc1S(N)(=O)=O)[C@@H](O)CN	12.2
**J01**	NS(=O)(=O)c1ccc(CCNCCc2ccccc2)cc1	1.6
**J03**	NS(=O)(=O)c1ccc(cc1)C1CCCNC1	3.1
**J05**	NCC1Cc2cccc(c2O1)-c1ccc(cc1F)S(N)(=O)=O	3.8
**J07**	NS(=O)(=O)c1ccc(CCNCc2ccsc2)cc1	5.9
**K01**	Cc1cc(cc(C)c1CN)S(N)(=O)=O	9.6
**K02**	Cc1ccc(CNCCc2ccc(cc2)S(N)(=O)=O)s1	5.2
**K03**	CC1CN(CCN1)S(=O)(=O)c1cccc(c1)S(N)(=O)=O	1.7
**K05**	NS(=O)(=O)c1ccc2CCNCCc2c1	2.1
**L01**	NS(=O)(=O)c1ccc(Cl)c2CNCCc12	5.4
**L03**	NS(=O)(=O)c1ccc(CCNCC(=O)NC2CC2)cc1	2.9
**M05**	NC1(Cc2ccc(cc2)S(N)(=O)=O)CC1	1.7
**M07**	CC(CNCCc1ccc(cc1)S(N)(=O)=O)c1ccccc1	7.1
**N01**	NS(=O)(=O)c1ccc(CCNC(=O)[C@@H]2CCCNC2)cc1	38.4
**N07**	NCCNC(=O)c1ccc(Cl)c(c1)S(N)(=O)=O	11.6
**O01**	CC(N)c1ccc(cc1)S(N)(=O)=O	12.4
**O03**	NC1CCc2ccc(cc12)S(N)(=O)=O	11.4
**O04**	NS(=O)(=O)c1ccc(CCNCc2ccccc2F)cc1	1.8
**O07**	NCCc1cccc(c1)S(N)(=O)=O	4.8
**P05**	NCCCc1ccc(cc1)S(N)(=O)=O	38.6
**P07**	CC1CNCCN1S(=O)(=O)c1cccc(c1)S(N)(=O)=O	1.8

7. Compounds

7.1 Synthesis of covalent warhead

3-(3-methyl-3*H*-diazirin-3-yl)propanoic acid (S5)[6]

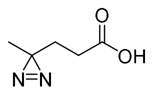

A solution of levulinic acid (5.00 g, 43.1 mmol) in 7 M ammonia in methanol (MeOH) (50 mL, 350 mmol) was stirred at 0 °C for 3 h. (Aminooxy)sulfonic acid (7.30 g, 64.6 mmol) was added slowly to the reaction mixture which was allowed to warm to room temperature for 18 h. Nitrogen was bubbled through the mixture for 1 h. The suspension was filtered and the white solid was washed with MeOH (1 × 30 mL). The filtrate was concentrated *in vacuo* to afford a yellow oil. The solid was dissolved in MeOH (40 mL) and triethylamine (18.0 mL, 129 mmol) was added. The solution was stirred at room temperature for 5 min before being cooled to 0 °C. Iodine pellets (12.0 g, 47.4 mmol) were added portionwise over 5 min and the solution was left to stir for 10 min at 0 °C. To the reaction mixture, 2 M aq. HCl (50 mL) and 10% (*w/v*) aq. sodium thiosulfate (90 mL) were added. The mixture was then extracted with ethyl acetate (EtOAc) (3 × 150 mL). The combined organic layers were dried (hydrophobic frit) before being concentrated *in vacuo* to afford an orange liquid. The liquid was taken up in a minimum amount of dichloromethane (CH_2Cl_2), filtered through cotton wool and purified by flash column chromatography (silica, 0–50% (3:1 (*v/v*) EtOAc:ethanol (EtOH)) in cyclohexane). The relevant fractions, identified by potassium permanganate staining, were combined and concentrated *in vacuo* to afford 3-(3-methyl-3*H*-diazirin-3-yl)propanoic acid (**S5**) (1.95 g, 15.2 mmol, 35% yield)[6] as a pale yellow liquid.

Appearance: yellow liquid. Infrared spectrum: ν_{max} (liquid): 2928, 1708, 1585, 1415, 1289, 1222, 918, 660, 470 cm^{-1}. ^{1}H NMR (400 MHz, $CDCl_3$) δ = 2.24 (t, *J*=7.6 Hz, 2 H) 1.73 (t, *J*=7.6 Hz, 2 H) 1.05 (s, 3 H). ^{13}C NMR (101 MHz, DMSO-d_6) δ = 173.2, 29.1, 28.2, 25.5, 19.2 ppm.

2,5-dioxopyrrolidin-1-yl 3-(3-methyl-3*H*-diazirin-3-yl)propanoate (1)

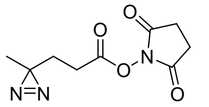

A solution of 3-(3-methyl-3*H*-diazirin-3-yl)propanoic acid (**S5**) (2.05 g, 16.0 mmol), 2-(2,5-dioxopyrrolidin-1-yl)-1,1,3,3-tetramethylisouronium tetrafluoroborate (5.30 g, 17.6 mmol) and *N,N*-dimethylpyridin-4-amine (2.15 g, 17.6 mmol) in acetonitrile (25 mL) was stirred at rt for 0.5 h. The reaction mixture was partitioned between EtOAc (50 mL) and water (50 mL). The organic layer was collected and the aqueous layer was extracted three times with EtOAc (3 × 50 mL). The combined organic layers were dried (hydrophobic frit) before being concentrated *in vacuo* to afford a yellow liquid. The liquid was dissolved in MeOH (25 mL) before being passed over a 10 g strong cation exchange (SCX) cartridge with methanol washings (1 × 100 mL). The filtrate was concentrated *in vacuo* to afford an off-white solid. The solid was purified by flash column chromatography (silica, 0–100% EtOAc in cyclohexane). The fractions were concentrated *in vacuo* to afford 2,5-dioxopyrrolidin-1-yl 3-(3-methyl-3*H*-diazirin-3-yl)propanoate (**1**) (2.04 g, 9.10 mmol, 57% yield) as a white solid.

Appearance: white solid. Melting point: 76.9–78.7 °C. Infrared spectrum: ν_{max} (solid): 2953, 1819, 1778, 1726, 1586, 1381, 1216, 1073, 898, 648 cm^{-1}. ^{1}H NMR (400 MHz, $CDCl_3$) δ = 2.84 (s, 4H), 2.56 - 2.48 (m, 2H), 1.85 - 1.77 (m, 2H), 1.08 (s, 3H) ppm. ^{13}C NMR (101 MHz, $CDCl_3$) δ = 168.9, 167.6, 29.5, 25.7, 25.6, 24.7, 19.5 ppm. HRMS: ($C_9H_{11}N_3O_4Na$) $[M+Na]^+$ requires 248.0647, found $[M+Na]^+$ 248.0647.

7.2 General procedure for the synthesis of primary sulfonamide hits

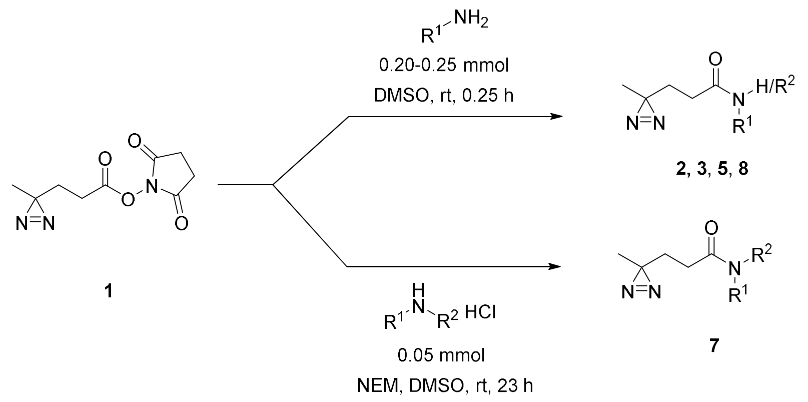

Scheme S1: Synthesis of selected hit PhABits.

Primary amines: A solution of compound **1** (1.1 eq.) and an amine (0.20-0.25 mmol) in DMSO (1-2 mL) was stirred at room temperature for 0.25 h.

Secondary amine: A solution of compound **1** (1.1 eq.), an amine.HCl salt (0.05 mmol) and *N*-ethylmorpholine (3 eq.) in DMSO (1 mL) was stirred at room temperature for 23 h.

The reaction mixtures were purified by MDAP (HpH_Method_B). The relevant fraction(s) were combined and concentrated *in vacuo*. The products were dried in a vacuum oven, heated at 40 °C, for 2-16 h to afford the desired products (**2**, **3**, **5**, **7** and **8**).

7.3 Primary sulfonamide hits

_N_-(2-chloro-6-methyl-4-sulfamoylbenzyl)-3-(3-methyl-3_H_-diazirin-3-yl)propanamide (2)

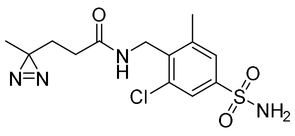

Following the general procedure outlined in **section 7.2** afforded *N*-(2-chloro-6-methyl-4-sulfamoylbenzyl)-3-(3-methyl-3*H*-diazirin-3-yl)propanamide (**2**) (32 mg, 0.093 mmol, 55%) as a white solid. Infrared spectrum: ν_{max} (solid): 3343, 3266, 1637, 1534, 1330, 1183, 1148, 915, 873, 733, 589 cm^{-1}. ^{1}H NMR (400 MHz, DMSO-d_6) δ = 8.06 (br t, *J*=4.9 Hz, 1H), 7.68 (d, *J*=1.5 Hz, 1H), 7.60 (d, *J*=1.5 Hz, 1H), 7.44 (s, 2H), 4.41 (d, *J*=4.9 Hz, 2H), 2.45 (s, 3H), 1.96 - 2.02 (m, 2H), 1.54 - 1.59 (m, 2H), 0.96 (s, 3H). ^{13}C NMR (151 MHz, DMSO-d_6) δ = 170.6, 144.1, 141.1, 137.7, 134.6, 125.7, 123.8, 37.7, 29.8, 29.3, 25.7, 19.7, 19.2. LC-MS (CSH~2min_HpH) t_r = 0.82 min, $[M-H]^-$ = 343.13, (100% purity). HRMS: ($C_{13}H_{17}N_4O_3SCl$) $[M+H]^+$ requires 345.0710, found $[M+H]^+$ 345.0779.

3-(3-methyl-3_H_-diazirin-3-yl)-_N_-(1-(3-sulfamoylphenyl)ethyl)propanamide (3)

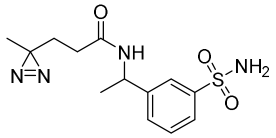

Following the general procedure outlined in **section 7.2** afforded 3-(3-methyl-3*H*-diazirin-3-yl)-*N*-(1-(3-sulfamoylphenyl)ethyl)propanamide (**3**) (44 mg, 0.142 mmol, 71%) as a colourless gum. Infrared spectrum: ν_{max} (solid): 3271, 3069, 2976, 2928, 1645, 1532, 1327, 1307, 1154, 896, 797, 692, 588, 509 cm^{-1}. ^{1}H NMR (400 MHz, DMSO-d_6) δ = 8.41 (d, *J*=7.2 Hz, 1H), 7.73 - 7.77 (m, 1H), 7.65 - 7.72 (m, 1H), 7.48 - 7.53 (m, 2H), 7.32 (s, 2H), 4.95 (quin, *J*=7.2 Hz, 1H), 1.96 - 2.09 (m, 2H), 1.57 (t, *J*=7.6 Hz, 2H), 1.36 (d, *J*=7.2 Hz, 3H), 0.97 (s, 3H). ^{13}C NMR (101 MHz, DMSO-d_6) δ = 169.9, 145.7, 144.1, 129.3, 128.8, 123.9, 122.9, 47.6, 29.7, 29.7, 25.7, 22.2, 19.3. LC-MS (CSH~2min_HpH) t_r = 0.74 min, $[M+H]^+$ = 310.99, (100% purity). HRMS: ($C_{13}H_{18}N_4O_3S$) $[M+H]^+$ requires 311.1100, found $[M+H]^+$ 311.1170.

3-(3-methyl-3*H*-diazirin-3-yl)-*N*-(3-(sulfamoylmethyl)benzyl)propanamide (5)

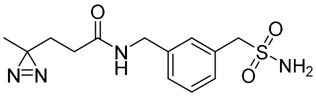

Following the general procedure outlined in **section 7.2** afforded 3-(3-methyl-3*H*-diazirin-3-yl)-*N*-(3-(sulfamoylmethyl)benzyl)propanamide (**5**) (10 mg, 0.032 mmol, 16%) as a white solid. Infrared spectrum: ν_{max} (solid): 3326, 1621, 1538, 1338, 1140, 894, 807, 702, 519, 479 cm^{-1}. ^{1}H NMR (400 MHz, DMSO-d_6) δ = 8.34 (br t, *J*=5.9 Hz, 1H), 7.29 - 7.35 (m, 1H), 7.21 - 7.27 (m, 3H), 6.81 (s, 2H), 4.26 (d, *J*=5.9 Hz, 2H), 4.23 (s, 2H), 2.01 - 2.07 (m, 2H), 1.58 - 1.63 (m, 2H), 0.99 (s, 3H). ^{13}C NMR (151 MHz, DMSO-d_6) δ = 170.6, 139.4, 130.7, 129.5, 129.2, 128.2, 126.7, 60.1, 41.9, 29.7, 29.6, 25.8, 19.3. LC-MS (CSH~2min_HpH) t_r = 0.70 min, $[M-H]^-$ = 309.16, (99% purity). HRMS: ($C_{13}H_{18}N_4O_3S$) $[M+H]^+$ requires 311.1100, found $[M+H]^+$ 311.1170.

3-methyl-2-(3-(3-methyl-3*H*-diazirin-3-yl)propanoyl)-1,2,3,4-tetrahydroisoquinoline-7-sulfonamide (7)

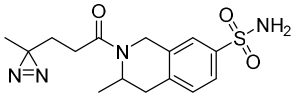

Following the general procedure outlined in **section 7.2** afforded 3-methyl-2-(3-(3-methyl-3*H*-diazirin-3-yl)propanoyl)-1,2,3,4-tetrahydroisoquinoline-7-sulfonamide (**7**) (12 mg, 0.036, 67%) as a clear gum. Infrared spectrum: ν_{max} (solid): 3215, 2970, 1619, 1429, 1328, 1152, 650, 567, 546 cm^{-1}. ^{1}H NMR (400 MHz, DMSO-d_6, 100 °C) δ = 7.72 (s, 1H), 7.69 (br d, *J*=8.3 Hz, 1H), 7.36 (d, *J*=8.3 Hz, 1H), 6.88 (br s, 2H), 4.93 (br d, ^{2}J=17.1 Hz, 1H), 4.69 (br s, 1H, NC*H*CH_3), 4.38 (br d, ^{2}J=17.1 Hz, 1H), 3.10 (br dd, *J*=16.3, 5.0 Hz, 1H), 2.75 (dd, *J*=16.3, 2.6 Hz, 1H), 2.25 - 2.43 (m, 2H), 1.68 (t, *J*=7.3 Hz, 2H), 1.07 (d, *J*=6.6 Hz, 3H), 1.04 (s, 3H). ^{13}C NMR (151 MHz, DMSO-d_6, rotamers observed) δ = 169.7, 169.5, 142.0, 142.0, 137.6, 136.8, 133.5, 133.4, 129.6, 129.5, 124.0, 123.7, 123.4, 123.3, 45.8, 43.0, 42.6, 34.5, 33.9, 29.3, 29.3, 27.5, 26.8, 25.8, 19.5, 19.4, 18.2, 17.5. LC-MS (CSH~2min_HpH) t_r = 0.85 min, $[M-H]^-$ = 335.14, (100% purity). HRMS: ($C_{15}H_{20}N_4O_3S$) $[M+H]^+$ requires 337.1256, found $[M+H]^+$ 337.1323.

3-(3-methyl-3*H*-diazirin-3-yl)-*N*-(4-sulfamoylphenethyl)propanamide (8)

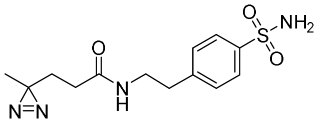

Following the general procedure outlined in **section 7.2** afforded 3-(3-methyl-3*H*-diazirin-3-yl)-*N*-(4-sulfamoylphenethyl)propanamide (**8**) (60 mg, 0.193, 77%) as a white solid. Infrared spectrum: ν_{max} (solid): 3304, 3091, 1627, 1539, 1338, 1158, 1097, 686, 591, 539 cm^{-1}. ^{1}H NMR (400 MHz, DMSO-d_6) δ ppm 7.94 (br t, *J*=7.1 Hz, 1H), 7.71 - 7.75 (m, 2H), 7.36 - 7.42 (m, 2H), 7.26 (s, 2H), 3.29 (q, *J*=7.1 Hz, 2H), 2.78 (t, *J*=7.1 Hz, 2H), 1.91 - 1.96 (m, 2H), 1.52 - 1.58 (m, 2H), 0.96 (s, 3H). ^{13}C NMR (151 MHz, DMSO-d_6) δ = 170.6, 143.6, 142.00, 129.0, 125.6 39.7, 34.7, 29.7, 29.7, 25.7, 19.3. LC-MS (CSH~2min_HpH) t_r = 0.70 min, $[M+H]^+$ = 310.94, (100% purity). HRMS: ($C_{13}H_{18}N_4O_3S$) $[M+H]^+$ requires 311.1100, found $[M+H]^+$ 311.1171.

8. References

1. E. K. Grant, D. J. Fallon, M. M. Hann, K. G. M. Fantom, C. Quinn, F. Zappacosta, R. S. Annan, C.-w. Chung, P. Bamborough, D. P. Dixon, P. Stacey, D. House, V. K. Patel, N. C. O. Tomkinson and J. Bush, *Angew. Chem. Int. Ed.*, 2020, **59**, 21096-21105.
2. A. J. Leo and D. Hoekman, *Perspect. Drug Discov.*, 2000, **18**, 19-38.
3. J. Szegezdi and F. Csizmadia, *233rd ACS National Meeting*, 2007, https://chemaxon.com/poster/a-method-for-calculating-the-pka-values-of-small-and-large-molecules.
4. M. Vargyas, J. Papp, F. Csizmadia, S. Csepregi, Á. Papp and P. Vadasz, *ChemAxon*, 2008.
5. D. Rogers and M. Hahn, *J. Chem. Inf. Model.*, 2010, **50**, 742-754.
6. M. R. Bond, H. Zhang, P. D. Vu and J. J. Kohler, *Nat. Protoc.*, 2009, **4**, 1044-1063.
